# Nanoconfined superionic water is a molecular superionic

**DOI:** 10.1126/sciadv.adz6392

**Published:** 2026-04-10

**Authors:** Samuel W. Coles, Amir Hajibabaei, Venkat Kapil, Xavier R. Advincula, Christoph Schran, Stephen J. Cox, Angelos Michaelides

**Affiliations:** ^1^ Yusuf Hamied Department of Chemistry, University of Cambridge, Lensfield Road, Cambridge CB2 1EW, UK.; ^2^ Lennard-Jones Centre, University of Cambridge, Trinity Ln, Cambridge CB2 1TN, UK.; ^3^ Department of Physics and Astronomy, University College London, 7-19 Gordon St, London WC1H 0AH, UK.; ^4^ Thomas Young Centre and London Centre for Nanotechnology, 9 Gordon St, London WC1H 0AH, UK.; ^5^ Cavendish Laboratory, Department of Physics, University of Cambridge, Cambridge CB3 0HE, UK.; ^6^ Department of Chemistry, Durham University, South Road, Durham DH1 3LE, UK.

## Abstract

Superionic ice, where water molecules dissociate into a lattice of oxygen ions and a rapidly diffusing “gas” of protons, represents a state of matter with broad implications for planetary interiors and energy applications. Recently, a nanoconfined superionic state of water has been predicted which, in contrast, is composed of intact water molecules. Here, we apply machine learning and electronic structure simulations to establish how nanoconfined water can be both molecular and superionic, providing more general insights into superionic behavior. Similar to bulk ice and other superionic materials, nanoconfined water conducts via concerted chain-like proton migrations, which cause the rapid propagation of defects. However, unlike other molecular phases of water, its exceptional conductivity arises from the activation of the Grotthuss mechanism by (i) low barriers to proton transfer and (ii) a flexible hydrogen bonded network. We propose that these are two key characteristics of fast ionic conduction in molecular superionics.

## Teaser

Nanoconfined superionic water is molecular, yet has an ionic conduction mechanism akin to that of inorganic superionics.

## INTRODUCTION

Solid-state ionics, the study of solid electrolytes, has its origins in Faraday’s discovery of the superionic phase of silver sulfide in the 1840s (*1*, *2*). Faraday observed a phase transition on heating silver sulfide. The presence of a phase transition between two solid-state phases at elevated temperature was expected. However, it was unexpected that the diffusion of silver ions in this higher-temperature solid phase exhibited an ionic conductivity equivalent to that of a liquid electrolyte. Later, scientists, drawing inspiration from electronic superconductivity, would term this phenomenon superionicity (*3*). Superionic behavior in chemically simple materials such as AgI and PbF_2_ occurs at impractically high temperatures. However, recently designed materials based on lithium lanthanum zirconium oxide (*4*, *5*), lithium germanium thiophosphate (*6*), and superionic polymers (*7*, *8*) are now being adopted for use in electrochemical devices.

Superionic water ice is one of the most widely studied and scientifically fascinating superionic materials. It is traditionally composed of fully dissociated water molecules involving a “gas” of rapidly diffusing protons within a relatively stationary lattice of oxygen ions (*9*–*13*). However, bulk superionic water phases (such as ice XVIII) form at the exceptional temperatures and pressures found in the cores of, e.g., Uranus and Neptune [~2000 K, ~55 GPa (*9*–*14*)]. Recently, a superionic state of water has been predicted to form at milder conditions when it is confined within subnanometer-wide slit pores (*15*). This nanoconfined superionic water is predicted to form at as low as 400 K and near the low GPa pressures naturally created within van der Waals bonded materials such as graphene (*16*). Similar behavior has also been observed in recent experiments of nanoconfined water in slit pores, where there is evidence of exceptionally high ionic conductivity (*17*). A superionic state of water at or near room temperature is a tantalizing prospect scientifically and technologically.

In contrast to bulk superionic water and conventional superionic materials, simulations suggest that nanoconfined water is composed of intact water molecules (*15*, *18*, *19*). However, as yet, there has been no comparison of the diffusive mechanism of this molecular system to more conventional superionic materials. In addition, it is unclear why it forms nor what “rules” govern the formation of superionic states under nanoconfinement. Here, we use the results of machine learning molecular dynamics simulations and electronic structure calculations to reveal the chemical and physical nature of superionicity under nanoconfinement and to define the rules which govern its function and behavior.

We confirm that nanoconfined water has a quasi-crystalline hexatic molecular structure, in sharp contrast to bulk superionic water (*15*), which is better described as a giant inorganic structure. Despite this difference, just as in conventional superionics, nanoconfined superionic water conducts via the rapid propagation of defects with a mechanism based on diffusive chains. In the case of nanoconfined superionic water, these defects are hydroxide and hydronium ions, which are the analogs of charge carrier vacancies and interstitial defects in conventional superionics. These defect ions can form in this neutral system via autoionization due to the short separation between oxygen atoms and exist at exceptionally high concentrations compared to bulk aqueous systems. This means that this system is both molecular and superionic and can be described as a molecular superionic. We show that the high ionic conductivity in nanoconfinement arises from facile proton transfer and hydrogen bond rearrangement (*20*, *21*), which are caused by short distances between oxygen atoms and dangling hydrogen bonds, respectively (*22*, *23*). Last, we consider how these insights can accelerate the discovery of other molecular superionic materials.

## RESULTS

### The contrast in structure and bonding between molecular and conventional superionic water

We begin by building on the work performed in prior studies characterizing the structures of nanoconfined (*15*, *18*, *19*) and bulk superionic water (*9*, *12*, *13*, *24*). We focus on bcc-ordered bulk superionic water because of its structural similarity to AgI and other fast-ion conductors (*25*). Results for fcc bulk superionic water, showing similar behavior, are given in the Supplementary Materials.

**Fig. 1.**
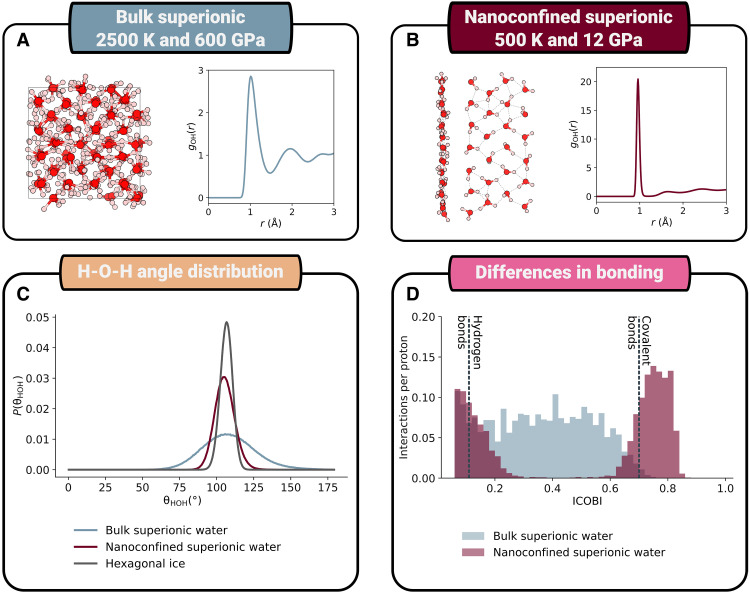
Bulk superionic water is not molecular, but nanoconfined superionic water is. (**A** and **B**) Representative structural snapshots (left) and plots of $g_{\text{OH}}\left(r\right)$ (right) for bulk and nanoconfined superionic water respectively. In the case of the nanoconfined system, both side and top views are shown of a selected portion of the unit cell. (**C**) Plots of the hydrogen-oxygen-hydrogen bond angles in the two phases as well as hexagonal ice at 250 K and 0.1 MPa. (**D**) Comparison of the chemical bonding in the two systems using an electronic structure descriptor, specifically the ICOBI index (see text for further details). The dotted lines on this panel reflect the average ICOBI values for the hydrogen bonds and covalent bonds in bulk hexagonal ice.

Figure 1 reveals a sharp contrast in the structure between nanoconfined superionic water and bulk superionic water. Specifically, in Fig. 1A, we report structural information at 2500 K and 600 GPa, corresponding to the region of stability of the bcc phase. In the snapshot shown on the left, we see that the structure has a clear periodic arrangement of both oxygen and hydrogen ions, with protons normally centered on the interstices in the periodic lattice. The radial distribution function (RDF) between protons and oxygens in this system, which is presented on the right, has a form comprising a broad first peak with a magnitude similar to an ion pair in a molten salt (*26*, *27*), followed by an oscillatory decay to a value of 1 with a periodicity derived from the periodic ordering in the crystalline lattice. This form is characteristic of that between mobile and framework ions in a conventional superionic material (for instance AgI, an exemplar superionic material, shown fig. S6) (*28*). Although differences exist between a conventional superionic and the bulk superionic water, they can both be described as ionically conductive inorganic crystals. This leads us to describe bulk superionic water as a conventional superionic material.

In contrast, if we consider these same descriptors for nanoconfined superionic water (Fig. 1B), then the structure is clearly molecular. First, it is clear on inspection of the structural snapshot that its constituent units are water molecules. A point that also leads to the sharp, narrow form of the first peak in $g_{\text{OH}}\left(r\right)$ caused by the strong molecular covalent bonds between oxygen and hydrogen.

The difference in nature between the two systems is made clearer still if we consider the H-O-H bond angle in the two superionic systems and hexagonal ice (0.1 MPa, 250 K) shown in Fig. 1C. In the case of nanoconfined superionic water, the bond angle distribution is narrow, similar to that of hexagonal ice and centered on 106.3°. This is characteristic of the strong constraint placed on the bond angle in molecular water. For bulk superionic water, however, the distribution is different with a much broader range of bond angles being observed. This relative lack of constraint emerges from the nonmolecular nature of the bonding in the phase, where the arrangement of protons is mainly templated by the geometry of the oxygen lattice as opposed to being driven by molecular geometry.

We have shown that the bulk and nanoconfined superionic water have fundamentally different structures. However, it is not clear whether this difference is correlated with an underlying difference in chemical bonding. To explore this, we use density functional theory (DFT) to compute the electronic structure of each system, and we then analyze this electronic structure with an approach known as the Integrated Crystal Orbital Bond Index (ICOBI) (*29*). ICOBI is one of many quantum chemical approaches for obtaining qualitative insights into bonding and is particularly useful when trying to distinguish ionic from covalent bonding. The value of ICOBI is a proxy for the covalency of a bond, with 1 being representative of a fully covalent interaction and 0 for an interaction where no covalency is observed (either due to a purely ionic interaction or when atoms do not interact at all). The main result from this analysis is shown in Fig. 1D, where we plot the distribution in ICOBI values for nanoconfined and bulk superionic water. The distribution for nanoconfined water consists of two peaks, which align with the values of the covalent and hydrogen bonds in hexagonal ice (shown as dotted lines). This is indicative of the molecular nature of nanoconfined superionic water. The plot for bulk superionic water is notably different. There is a broad range of ICOBI values continuously from 0.7 to 0, reflective of a system composed of thermally fluctuating protons located primarily on interstitial sites in the bcc lattice. The form of this distribution is similar to that for the conventional superionic material silver iodide, which is shown in the Supplementary Materials (fig. S5). Therefore, we can see that bulk superionic water is best understood as adopting an inorganic crystalline structure, with a degree of covalent character (*13*, *24*). This, it should be noted, is not universal for all planetary superionic ices. For instance, at substantially lower temperatures (~700 K with a pressure of 70 GPa), ammonia has been predicted to form a superionic state consisting primarily of molecular NH_2_^−^ and NH_4_^+^ ions (*30*, *31*). This superionic ammonia ice and nanoconfined superionic water are both superionic and molecular and therefore represent a distinct class of superionic materials; we term this class molecular superionics.

### Defect migration and correlated proton transport in nanoconfined water

Materials have conventionally been considered superionic if they have a conductivity that exceeds 0.1 S/cm (*32*). At 500 K and 12 GPa, we find nanoconfined superionic water to have a conductivity of 0.15 to 0.17 S/cm, making it superionic by this definition. In addition, in the broader field of solid-state ionics, criteria for diffusive mechanisms in superionic materials have been proposed (*28*, *33*, *34*). Specifically, it has been suggested that, in superionic materials, diffusion occurs (i) via defects and (ii) correlated ion migrations. We now explore whether nanoconfined superionic water meets these descriptors.

**Fig. 2.**
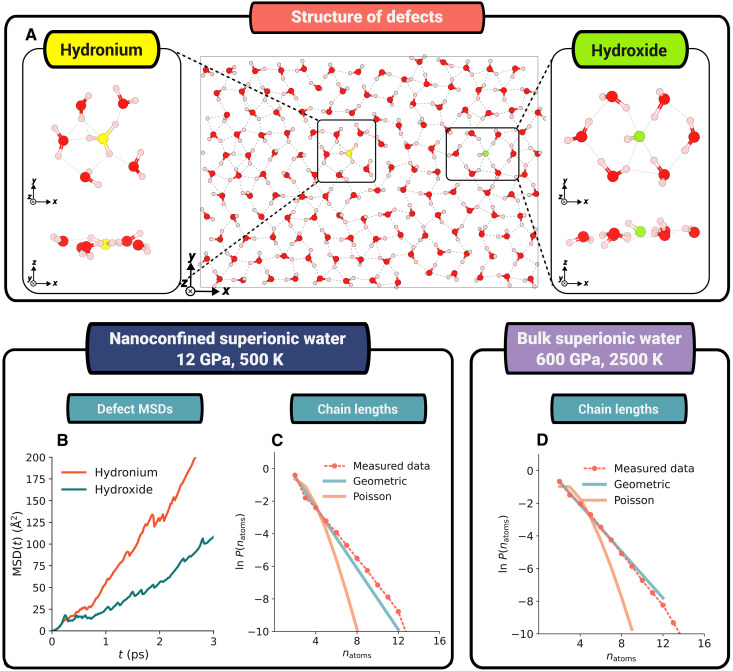
Both nanoconfined superionic water and bulk superionic water undergo chain-like defect-based diffusion. (**A**) Snapshot of a nanoconfined system with both a hydroxide ion (oxygen atom is shown in green) and a hydronium ion (oxygen atom is shown in yellow) with zoomed in snapshots of the solvated structures of these ions. (**B**) MSD plots of the defects. (**C** and **D**) Probability of diffusive chains consisting of a certain number of hydrogen atoms in nanoconfined and bulk superionic water, respectively.

The conduction mechanisms in conventional superionic conductors often occur through the propagation of defects, specifically either interstitial ions or charge carrier vacancies (*34*, *35*). During superionic conduction, these defects propagate through the system rapidly at a rate far faster than that of the individual charge carriers. In nanoconfined superionic water, the defects are solvated hydroxide and hydronium ions. These are shown in Fig. 2A and are direct analogs of the vacancies and interstitials that constitute the so-called Frenkel pairs in conventional superionic materials (*36*). These defect pairs form spontaneously by the transfer of a proton from one water molecule to another. In some cases, the proton immediately transfers back leading to the annihilation of these defects. However, when this does not happen, they propagate through space via the Grotthuss mechanism (*37*, *38*). This causes them to become separated like they are in the snapshot in Fig. 2A. Eventually, each defect will meet another alternatively charged defect and, if proton transfer occurs, annihilate. The average mean squared displacement (MSD) of each defect species in Fig. 2B shows them both to be diffusive, with the hydronium defects diffusing faster than the hydroxide. The faster hydronium defect diffusion observed here is consistent with simulations of dilute defects in bulk water (*39*).

In bulk superionic water, the defect chemistry is based on conventional crystalline interstitials and vacancies, not hydroxide and hydronium ions. It is not appropriate to describe the oxygens as hydroxides and hydroniums due to the bonding of protons to multiple oxygen atoms. When we attempt to apply this nomenclature (fig. S10), some oxygens are arbitrarily assigned as O^2−^ and H_4_O^2+^. The defect chemistry we observe in nanoconfined superionic water is far more reminiscent of conduction in acids and bases (*40*, *41*). With the exceptionally low *pKw *of nanoconfined superionic water—ranging from 3 to 1.5 with temperature (discussed in section S4)—signifying concurrent concentrations of hydroxide and hydronium ions equivalent to strong bases and acids, respectively.

Let us consider now correlated ion migrations, as opposed to isolated hoping events consisting of the movement of a single ion (*33*, *42*, *43*). In Fig. 2, we report the probability that a diffusive chain of proton hops in nanoconfined superionic water (Fig. 2C) and bulk superionic water (Fig. 2D) consists of a certain number of atoms. If the diffusive events were independent of one another and chains were forming via uncorrelated hops, we would expect these logarithmic graphs to follow Poisson distributions. It is clear, however, that they both have a geometric (straight line) form, indicating the presence of a chain-like diffusion mechanism comprising correlated hops. This chain-like diffusion is also observed for silver iodide (fig. S7) and multiple other superionic materials (*42*–*44*). Thus, like other superionic materials, nanoconfined water undergoes correlated ion motion. In contrast, however, the system remains molecular and the motion Grotthuss-like. It is therefore clear that nanoconfined superionic water is both intrinsically superionic and intrinsically different from other superionic materials. This further motivates our classification of it as a molecular superionic.

**Fig. 3.**
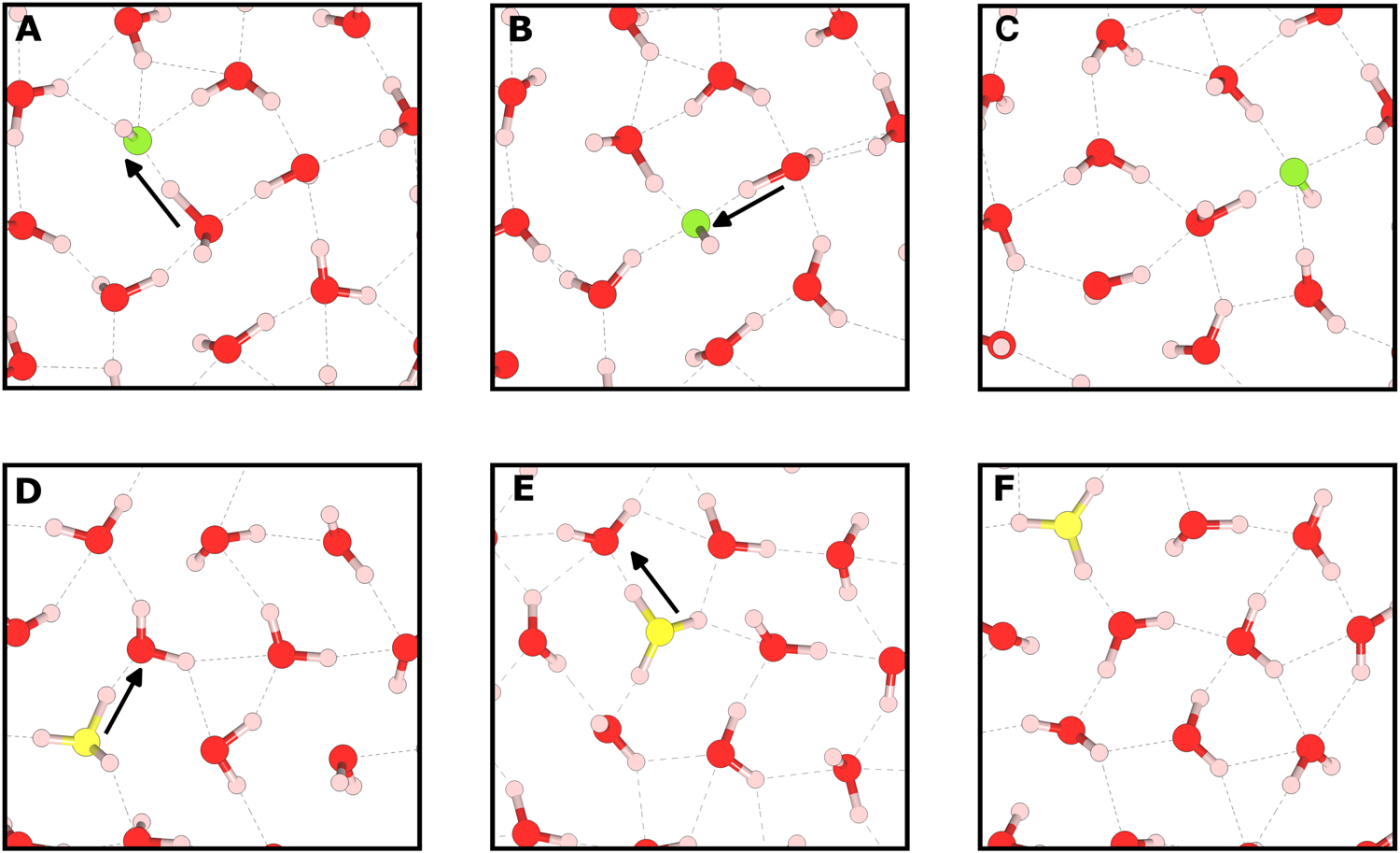
Snapshots from our molecular dynamics trajectories showing examples of defect motion in nanoconfined water. (**A** to **C**) Hydroxide diffusion via Grotthuss hopping. (**D** to **F**) Hydronium diffusion via Grotthuss hopping. The hydroxide ion is shown in green, and the hydronium ion is shown in yellow. For clarity, only a small portion of the unit cell is shown in these snapshots.

The Grotthuss-like nature of defect diffusion is illustrated in Fig. 3, where individual proton hopping events are seen to propagate proton defects through the system. The cumulative effect of many such hops in sequence leads to long-range defect diffusion, resulting in the large defect MSDs observed in Fig. 2B. This behavior is directly captured in movies S1 and S2.

### Low proton transfer barriers and hydrogen bond network flexibility as design principles

**Fig. 4.**
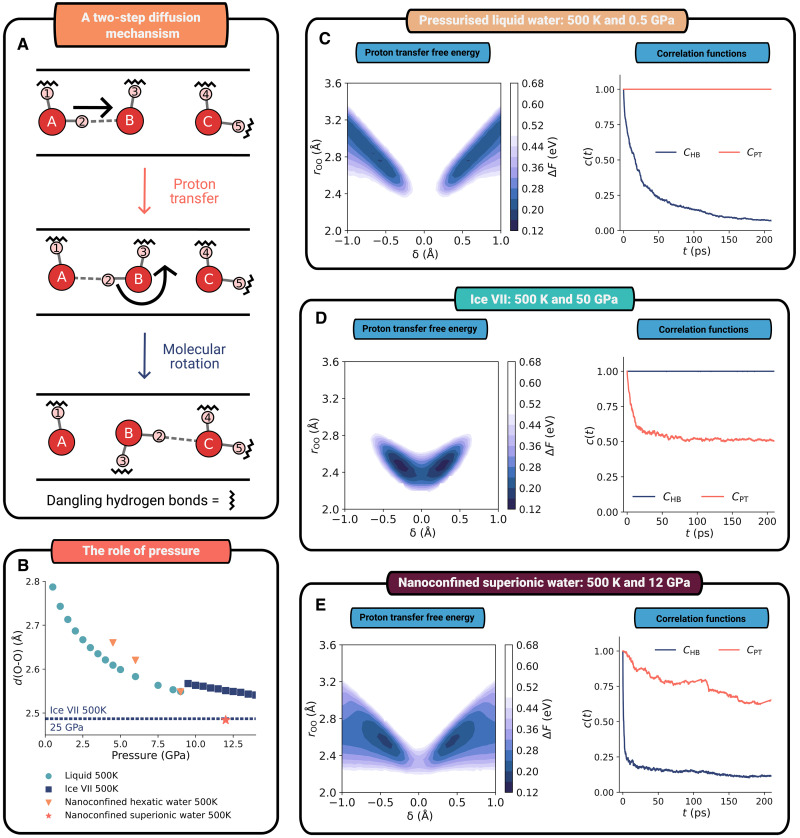
Closer separation of oxygen atoms and hydrogen bond network flexibility enables a two-step proton diffusion mechanism in nanoconfined superionic water. (**A**) A simplified scheme of the proton diffusion mechanism in nanoconfined superionic water. (**B**) Influence of pressure on the separations between oxygens in liquid water, ice VII, and nanoconfined superionic water. (**C** to **E**) Correlation functions and free energies of pressurized liquid water, ice VII, and nanoconfined superionic water. For each material, we present descriptors that show its ability to undergo the two-step diffusion process shown in (A). On the left-hand side, free energies for proton transfer are plotted as a function of oxygen-oxygen separation ($r_{\text{OO}}$) and the location of the proton along the oxygen-oxygen axis (∂). On the right-hand side, we present the correlation functions $C_{\text{HB}}$ and $C_{\text{PT}}$ (described in the main text and Materials and Methods) for each of the three phases.

The prominence of the chain-like Grotthuss mechanism in nanoconfined superionic water’s diffusion mechanism may at first seem expected, since this is the conventional mechanism of proton conduction in molecular water-based systems. However, there is—as we would expect from our structural analysis—an alternative chain-like mechanism of proton conduction in the nonmolecular bulk superionic ice. As superionicity is not observed in any molecular water ices (*45*, *46*), there must be a specific feature of nanoconfined superionic water—as well as the conductive molecular ammonia ices—which allows Grotthuss to drive superionic conduction. Drawing on the commonality with conventional superionic conductors, we can propose two key features that are necessary for a material to be conductive. First, the barrier for charge carrier motion is sufficiently close to thermal energy that charge carriers can migrate (*33*). Second, there are diffusive pathways in the system that allow an ion to move in a direction other than back and forth between neighboring sites (*42*) (i.e., to do more than rattle within a single O-O pair). In Fig. 4, we explore how both criteria are met in nanoconfined superionic water in contrast with other neutral molecular water-based systems. We show that when both criteria are not met, ionic conduction is suppressed.

First, we investigate the free energy barrier to proton transfer for each system. These are shown in the left of Fig. 4 (C to E), where we plot a two-dimensional free energy surface of oxygen-oxygen separation ($r_{\text{OO}}$) versus proton distance from the oxygen-oxygen midpoint (δ). This is a widely used coordinate system for exploring proton transfer in aqueous systems (*39*, *41*, *47*), with the midpoint (δ = 0) representing the transition state for proton transfer (see Materials and Methods for further details). In the pressurized water plot (Fig. 4C, left), there is a considerable barrier to proton transfer.

In nanoconfined superionic water, the barrier is decreased as shown by the substantially lower saddle point in the free energy plot in the left of Fig. 4E, indicating the energetic favorability of proton transfer in this phase. The pressure-driven lowering of this barrier is critical to the phase transition to the superionic phase from the nonconductive hexatic phase and has previously been described by Kapil *et al.* (*15*) and Ravindra *et al.* (*19*). This reduced barrier directly enables sustained sequences of proton hopping events, which collectively give rise to long-range defect diffusion and finite ionic conductivity. Consequently, the proton transfer mechanism itself determines the conductive character of the superionic phase, and suppressing conduction necessarily corresponds to leaving this regime rather than tuning conductivity independently within it. Bulk superionic water shows a similar effect, but it involves a much more marked structural change—from nonconductive molecular ice (ice VII) to a conductive, inorganic-like structure—than the small barrier reduction seen here.

This decrease in barrier height arises from a pressure-driven decrease in separation between oxygens. In Fig. 4B, we observe this decrease in the separation between oxygens with pressure in nanoconfined superionic water. However, we also observe that bulk pressurized water and ice VII do not reach similar separations in the same pressure range. This suggests that the close separation—and with it the decreased proton transfer barrier—emerges from the twin effects of pressure and a nanoconfinement-induced change in the relationship between separation and the applied pressure.

However, enabling proton transfer—the motion of protons between neighboring molecules—is not sufficient to activate diffusion in a molecular water-based material. Ice VII can reach oxygen separations similar to nanoconfined water at pressures more than 25 GPa. At these separations, there is a high degree of proton transfer—as we can observe in the free energy plot on the left of Fig. 4D (at elevated temperatures in excess of 500 K). Despite this, ice VII remains an exceptionally poor conductor under these conditions, with a conductivity six orders of magnitude lower than nanoconfined superionic water (*46*). The origin of this difference lies in whether there are diffusive pathways in the two systems. In an inorganic superionic conductor, each site within this system is connected to multiple others, which leads to extended interconnected diffusive pathways. In contrast, in a molecular water-based system, intermolecular proton transfer occurs along hydrogen bonds. Proton transfer alone, therefore, cannot lead to proton diffusion (the long-range mass transport of protons), because after a proton transfer event, there is nowhere for a proton to go other than the direction it came. Drawing inspiration from studies of proton transfer in acidic systems (*20*, *21*, *38*, *40*) and the recent work by Gomez *et al.* (*48*) and Das *et al.* (*23*), we propose the two-step mechanism for diffusion in nanoconfined superionic water shown in Fig. 4A. In this mechanism, proton transfer is followed by molecular rotation, leading to a change in hydrogen bonding, after which any additional intermolecular proton transfer can occur to a third oxygen molecule. Repetition of this process opens diffusive pathways in the system allowing proton diffusion to occur.

In the rightmost plot of Fig. 4 (C to E), we investigate whether this two-step mechanism is possible in ice VII, pressurized water, and nanoconfined superionic water. Taking inspiration from prior studies of proton transfer (*21*, *49*), we define two correlation functions $C_{\text{PT}}\left(t\right)$ and $C_{\text{HB}}\left(t\right)$ (which are defined mathematically in Materials and Methods). $C_{\text{PT}}\left(t\right)$ has a value of 1 when a proton is the nearest neighbor to the same oxygen at time *t* as it was at time 0 and a value of 0 otherwise. It describes the rate of change in covalent bonding and can be viewed as a proxy for proton transfer. $C_{\text{HB}}\left(t\right)$ is 1 if the two nearest-neighbor oxygens to each proton are the same at time *t* and time 0 and has a value 0 otherwise. It is a proxy for the rearrangement of the hydrogen bond network and does not change in the event of proton transfer (a rattling event). The proton transfer correlation function [$C_{\text{PT}}\left(t\right)$] can be related to the previously discussed proton transfer–based free energy plots on the left side of Fig. 4 (C to E).

For pressurized liquid water at 500 K (Fig. 4C), we observe the familiar behavior of ambient liquid water. The hydrogen bond network, of course, rearranges rapidly which leads to the decay in $C_{\text{HB}}\left(t\right)$ in the correlation plot in Fig. 4C. Water molecules are, however, too well separated leading to a high barrier to proton transfer—as can be observed in the central plot. This leads $C_{\text{PT}}\left(t\right)$ to maintain a value of 1, yielding the familiar limited conductivity of pure water.

For ice VII (Fig. 4D), in the rightmost plot, we see prompt decay of $C_{\text{PT}}\left(t\right)$ to a value of 0.5 on a picosecond timescale, indicating the presence of proton transfer. This is consistent with the relatively low free energy barrier for proton transfer shown on the left of Fig. 4D. However, $C_{\text{HB}}\left(t\right)$ maintains a constant value of 1 throughout the length of this simulation. This lack of variance in $C_{\text{HB}}\left(t\right)$ arises from a persistent hydrogen bonding network, presumably because of the saturated nature of the hydrogen bonding network in ice VII. Thus, the full two-step mechanism of proton diffusion does not occur, and again, this system is not conductive.

We therefore conclude that superionic transport in nanoconfined water arises because the reduced dimensionality places the system in a “Goldilocks” regime, in which proton transfer barriers are sufficiently low while the hydrogen bond network remains flexible enough to support Grotthuss-type proton transport. This balance is not realized in bulk liquid water, where the fully three-dimensional environment inhibits sustained proton hopping, nor in bulk superionic ice, where a rigid three-dimensional lattice suppresses molecular reorganization and long-range defect propagation. We observe decay in both $C_{\text{PT}}\left(t\right)$ and $C_{\text{HB}}\left(t\right)$ (Fig. 4E), revealing both proton transfer and hydrogen bond network rearrangement in the system and that the two-step mechanism of proton diffusion is viable. We have already noted the close separation of oxygens in nanoconfined superionic water, which enables proton transfer to occur. We suggest that the reordering of the hydrogen bonding network arises from the presence of dangling hydrogen bonds induced by nanoconfinement (*50*). Such dangling hydrogen bonds have recently been proposed by Das *et al.* (*23*) as a microscopic mechanism enabling enhanced molecular diffusion under extreme nanoconfinement, consistent with the transport behavior observed here. This means that there are nonhydrogen-bonded hydrogen atoms within the system, which mainly, although not exclusively, sit above or below the plane of oxygen atoms. This makes the hydrogen bonding network more flexible, facilitating molecular rotation and proton conduction.

## DISCUSSION

In summary, nanoconfined superionic water has a different structure from bulk superionic water and inorganic superionic materials. In contrast with these other superionic materials, it is composed of molecules. Despite these structural differences, it still conducts protons via the rapid propagation of defects—specifically hydroxide and hydronium ions—with a chain-like diffusion mechanism. We, therefore, conclude that nanoconfined superionic water is a molecular superionic.

The possibility of inducing superionicity in ice VII has previously been discussed (*45*, *46*). However, no molecular phase of pure water has been observed to exhibit superionicity under equilibrium conditions either experimentally or computationally (*45*). We have shown that nanoconfined superionic water’s unique conductivity is characterized by the presence of both proton transfer and a constantly rearranging hydrogen bond network. Proton transfer occurs primarily because of the closer separation between oxygen atoms at the elevated pressure at which the phase forms. The constant rearrangement of the hydrogen bond network is made possible by the increased flexibility arising from the lower number of hydrogen bonds formed by each water molecule and the associated presence of dangling hydrogen bonds (*23*). In this water-based nanoconfined superionic material, this conductivity occurs via the Grotthuss mechanism. The previously mentioned conductive molecular ammonia phase, which exists at similar temperatures and pressures to ice VII (*30*, *31*), can be viewed as a bulk crystalline molecular superionic. The difference in conductivity between this phase and ice VII is in ammonia’s molecular structure. Each ammonia molecule can donate three hydrogen bonds but can only accept one. This means that, in this dense ammonia-based molecular crystal, there will always be dangling hydrogen bonds in contrast to pristine water ice phases which have saturated hydrogen bond networks (although a similar effect could be obtained by the wholesale incorporation of defects). This also allows these ammonia-based crystalline materials to behave as molecular superionics. Just as in nanoconfined superionic water, conduction in these crystals is correlated with large amounts of disproportionation (in this case, to form NH_4_^+^ and NH_2_^−^) and a huge increase in the rotation of individual molecules in this superionic phase compared to nonconductive phases (*30*). The similarities in behavior between this ammonia-based phase and nanoconfined superionic water suggest that molecular superionicity is, in general, characterized by the presence of both a low-charge carrier barrier and a hydrogen bonding network, which actively rearranges, enabling long-range diffusion of charge carriers.

Let us now discuss how molecular superionicity might be observed in other systems, particularly under conditions closer to room temperature and pressure. First, mixtures of hydrogen bonding molecules are likely to be a promising avenue for further exploration. In these systems, disorder, and possibly variations in pH, could introduce both the dangling hydrogen bonds and intrinsic defects necessary to induce molecular superionicity. Second, a similar strategy of materials discovery that was used to discover both nanoconfined superionic water (*15*) and superionic molecular ammonia (*30*, *31*) could be applied to predict superionic phases in nanoconfinement which form at conditions closer to ambient conditions. This could involve the exploration of different combinations of hydrogen bonded molecules and different confining materials. Such systems are promising because they are now readily amenable to experimental investigation and can potentially support superionic states at milder conditions.

A third and likely initially simpler route would be to induce molecular superionicity at interfaces or within porous frameworks at reduced or ambient pressure. Reactive interfaces can introduce dangling hydrogen bonds in the same manner as nanoconfinement, so it would only be necessary for the specific interface to template a reduction in the separation of oxygen atoms for both of our proposed criteria for molecular superionicity. Encouragingly, this templating effect has already been observed at the reactive interfaces of metals and oxides where water strongly absorbs at certain sites, creating interfacial regions composed of a mixture of water molecules and hydroxide ions (*47*, *51*). This third approach would create a localized superionicity, not a superionic bulk material. This would bring with it a fine control of localized conductivity. This suggests that molecular superionicity could be a helpful tool in nanoscale devices and the control of sensitive surface-based aqueous chemical processes.

Last, we note on a broader point that the similarities and differences between conventional and molecular superionics described here are substantial. Here, we have discussed three different definitions of superionicity. That all three of them apply to both conventional superionics (*35*) and nanoconfined superionic water suggests that high conductivity, chain-like diffusion, and the presence of highly diffusive defects can be viewed as transferable criteria of superionicity

## MATERIALS AND METHODS

### Potentials and dynamics

We begin by discussing the potentials and settings used for the molecular dynamics simulations in this paper. Detailed descriptions of the specific systems modeled can be found in the Supplementary Materials.

#### Nanoconfined superionic water

The methodology of this paper was built around machine-learned molecular dynamics of bulk and nanoconfined water. The machine learning potential used to model nanoconfined water was developed by Kapil *et al.* (*15*) and is a neural network potential fit to a training set of calculations at the revPBE0-D3 level of theory, based on the procedure described by Schran *et al.* (*52*). This functional was chosen for its demonstrated quantitative agreement with high-level reference methods for the lattice energies of bulk and nanoconfined ice phases (*15*, *53*), proton transfer barriers in nanoconfined water (*19*), and the strength of water-carbon interactions (*54*). Data presented in this paper for nanoconfined superionic water are taken from simulations of the canonical ensemble with a time step of 0.25 fs, with a Nosé-Hoover thermostat and damping parameter of 20 fs. This simulation is run at the average density from a prior calculation in an NP*_xy_*T ensemble, where the barostat only acts in the *xy* dimension. As in the prior study by Kapil *et al.* (*55*), simulations were performed using i-PI 2.0 with interatomic forces calculated using the n2p2 lammps library (*56*). As in previous studies of nanoconfined superionic water, confinement is modeled implicitly using a Morse potential, fitted to recover the interactions obtained in quantum Monte Carlo simulations of water on graphene (*15*), with the forces arising from this potential being calculated using ASE (*57*). This confining potential is oriented such that it is uniform in the *xy* plane. A full structural description of the system is provided in the Supplementary Materials (fig. S1). Simulations were run between 1 and 2 ns in length depending on temperature. Extensive validations of the neural network potential can be found in the original publication of Kapil *et al.* (*15*).

#### Bulk ices: Ice VII and bulk superionic water

Simulations of ice VII and bulk superionic water were performed using a neural network potential (*58*) previously used and parameterized by Cheng *et al.* (*12*) using a training set of DFT calculations at the PBE-D3 level of theory, which has been applied previously to the study of bulk superionic ices (*12*) and high-pressure molecular ices (*59*). Simulations were run with the lammps molecular dynamics software (*60*), with the neural network potential implemented using the n2p2 lammps library (*56*). Simulations were run with a 0.25-fs time step. A two-step procedure was followed with an initial isotropic NPT (fixed pressure) run of 1 ns at a target temperature being used to calculate the equilibrium volume of the cell with a further 1-ns NVT (fixed volume) run performed to calculate structural details. All simulations were run with a temperature damping parameter of 20 fs and a pressure damping parameter of 200 fs within the Nosé-Hoover thermostat and barostat.

### Electronic structure calculations, defect tracking, and workflow

DFT calculations were performed using the VASP DFT software (*61*–*63*) using projector augmented wave potentials, the PBE exchange-correlation functional (*64*), and a 700-eV plane wave cutoff. ICOBI values were calculated from single-point VASP calculations using the LOBSTER software (*65*), with the pbeVaspFit2015 basis set. The structures considered were taken from the neural network potential molecular dynamics simulations and for the data reported in Fig. 1. Ten structures chosen at equal intervals throughout the trajectory from which the associated RDF was calculated were used.

### Analytical methods

Identification of defects and assignment of protons to oxygen atoms in the nanoconfined system is performed via Voronoi tessellation. This is appropriate as the highly molecular nature of the system means that most protons are closest to a single oxygen ion with which they are covalently bonded. The codes for this and other simple analyses use workflow built on the pymatgen (*66*), ASE (*57*), vasppy (*67*), kinisi (*68*, *69*), and numpy (*70*) scientific python libraries. Nanoconfined RDFs are subject to the same finite size correction described by Fong *et al.* (*71*).

### Calculation of chain lengths

The calculation of chain lengths follows the established methods used in the study of diffusive chains in the field of solid electrolytes (*43*, *44*) based on historic studies of glasses (*72*). Chains are identified on a pairwise basis where one atom—in our case limited to the hydrogen atoms in the system—has moved away from its initial site and been replaced by another. This is defined mathematically for atoms with positions $r_{i}$ and $r_{j}$ at time *t* as

$$\text{min}\left[\|r_{i}\left(t+\Delta t\right)-r_{j}\left(t\right)\|,\|r_{j}\left(t+\Delta t\right)-r_{i}\left(t\right)\|\right]<\delta$$ (1)

for a time window Δ*t* and a spatial cutoff δ. For both systems, we use Δ*t* values of 100 ps and a δ value of 1.75 Å. The latter value is taken to be less than the separation between physical sites in each system, which is the analog to the hard sphere radius in the simple glasses for which these methods were initially defined. The code used to calculate these chains is adapted from previous work (*73*).

### Correlation functions

The correlation function $C_{\text{PT}}\left(t\right)$ is defined as

$$C_{\text{PT}}\left(t\right)=\langle C_{\mathrm{ij}}\left(0\right)C_{\mathrm{ij}}\left(t\right)\rangle$$ (2)

where $C_{\mathrm{ij}}$ is 1 where an oxygen *i* is the nearest neighbor to hydrogen atom *j* (a proxy for a covalent bond between the two atoms) and 0 otherwise. This creates a correlation function for changes in covalent bonding. While $C_{\text{HB}}\left(t\right)$ is defined as

$$C_{\text{HB}}\left(t\right)=\langle C_{\mathrm{ij}}\left(0\right)C_{\mathrm{ij}}\left(t\right)H_{\mathrm{ik}}\left(0\right)H_{\mathrm{ik}}\left(t\right)+C_{\mathrm{ij}}\left(0\right)C_{\mathrm{ik}}\left(t\right)H_{\mathrm{ik}}\left(0\right)H_{\mathrm{ij}}\left(t\right)\rangle$$ (3)

where $H_{\mathrm{ij}}$ is 1 when oxygen *i* is next nearest neighbor to hydrogen atom *j* and 0 otherwise, and *k* is the index of an additional hydrogen atom. This equation can be viewed as a proxy for hydrogen bonding. Correlation functions were calculated using multiple time origins.

### Proton transfer free energy

In Fig. 4, we present plots of free energy of proton transfer: pressurized water, ice VII, and nanoconfined superionic water to undergo proton transfer and molecular rotations (the two components of our two-step mechanism). For proton transfer, we use a two-dimensional plot of free energy related to two variables δ and $r_{\text{OO}}$, which has been widely used in the literature (*39*, *41*, *47*, *51*). δ, the proton transfer coordinate, is defined for a proton located at $r_{H}$ between two oxygen atoms located at $r_{O_{1}}$ and $r_{O_{1}}$ and is mathematically defined as

$$\delta =\sqrt{{\left(r_{H}-r_{O_{1}}\right)}^{2}}-\sqrt{{\left(r_{H}-r_{O_{1}}\right)}^{2}}$$ (4)

This means that δ has a value of 0 for a proton perfectly equidistant between two oxygens. The separation between the two oxygens $r_{\text{OO}}$ is calculated as

$$r_{\text{OO}}=\sqrt{{\left(r_{O_{2}}-r_{O_{1}}\right)}^{2}}$$ (5)

The free energy relative to these variables is then calculated as

$$F\left(\partial ,r_{\text{OO}}\right)=-k_{B}\text{ln}P\left(\partial ,r_{\text{OO}}\right)$$ (6)

## Supplementary Materials

### The PDF file includes:

Supplementary Text Figs. S1 to S19 Table S1 Legends for movies S1 and S2 References
