# Supplementary material for: Nanoconfined superionic water is a molecular superionic
Source: Sci Adv. 2026 Apr 10;12(15):eadz6392. doi: 10.1126/sciadv.adz6392 (PMC13068076; doi:10.1126/sciadv.adz6392)
Supplement: Supplementary file 1 — Supplementary Text Figs. S1 to S19 Table S1 Legends for movies S1 and S2 References [file sciadv.adz6392_sm.pdf]

Science Advances
AAAS

Supplementary Materials for

Nanoconfined superionic water is a molecular superionic

Samuel W. Coles *et al.*

Corresponding author: Samuel W. Coles, swc46@cam.ac.uk; Angelos Michaelides, am452@cam.ac.uk

Sci. Adv. **12**, eadz6392 (2026)
DOI: 10.1126/sciadv.adz6392

The PDF file includes:

Supplementary Text
Figs. S1 to S19
Table S1
Legends for movies S1 and S2
References

Other Supplementary Material for this manuscript includes the following:

Movies S1 and S2

S1: Structural description of studied systems

S1.1: Nanoconfined superionic water

The setup used to study nanoconfined water in this paper is based on that used in Ref. (*15*). All structures in studies of nanoconfined superionic water have the form shown in Fig. S1,. Water molecules occupy a periodic space in the xy plane. In the z direction, an additional Morse potential is added to simulate the confining effect of graphene in a nanoslit of roughly 5 Å of the overall 28.33 Å of space in the z dimension. The remainder of space in the z dimension functioning as a vacuum slab greater than the cutoff of the neural network potential to prevent interaction with periodic images in that dimension.

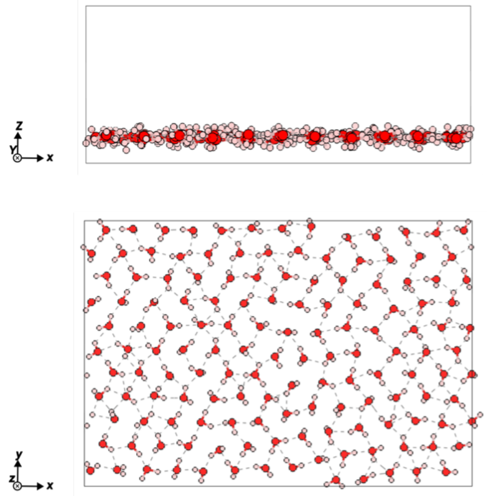

Figure S1: **Structural plans of the nanoconfined system of 144 water molecules, where oxygen atoms are shown in red and hydrogens in pink.** Water molecules are confined by an additional Morse potential in the z dimension, which confines the water molecules to a roughly 5 Å slit.

In general, simulations are performed in the NVT ensemble of 35.72 Å in the x dimension and 24.46 Å in the y dimension. The data from nanoconfined superionic water in Fig. 3B are generated from simulations previously run for Ref. (*19*). These simulations were run using an $NP_{xy}T$ ensemble (with the z dimension fixed with no change in the width of either the confining potential or vacuum slab).

S1.2: Bulk superionic water

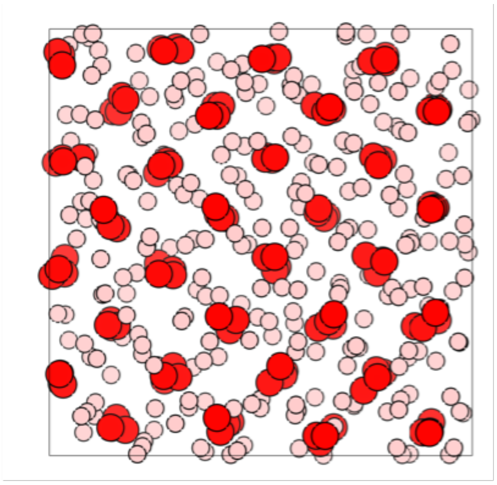

Figure S2: **The structure of the bulk superionic water used in the production of Fig. 1 in the main paper.** The structure consists of a bcc oxygen lattice. In the initial structure, protons are placed in locations such that molecular units could reasonably form. In this structure, oxygens are shown in red and hydrogens in pink.

Simulations and calculations of bulk superionic water are performed using the structure in Fig. S2. This structure has dimensions 9.72 Å × 9.81 Å × 9.81 Å and contains 384 atoms. The small size of the cell was chosen so that ICOBI calculations and structural descriptors could be calculated from the same cell. A doubling of the cell size leads to negligible change in structure as evidenced by the similarities in the form of $g_{\mathrm{OH}}(r)$ in Fig. S3.

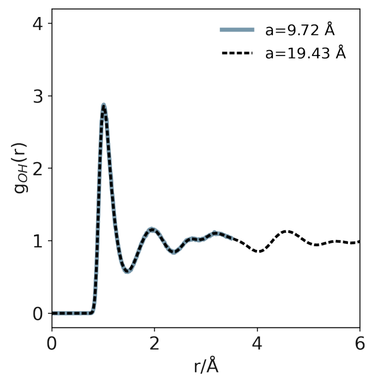

Figure S3: **Exploring finite size effects using a reproduction of the radial distribution function of bulk water from Fig. 1 in the main paper**. This figure shows the same result for the small cell used in the main paper and a $2 \times 2 \times 2$ expansion of that cell.

S1.3: Ice VII and pressurized water

Simulations and calculations of Ice VII were performed in a near-orthorhombic cell formed by a $6 \times 6 \times 4$ expansion of the ice VII primitive cell (as shown in Fig. S4). Simulations are performed in the NPT ensemble for the data points used to calculate the distances between oxygens. The calculations of correlation functions are performed in an NVT ensemble with a cell that has the same volume as the equilibrium volume from the 50 GPa run. This results in a cell with box side lengths 17.39 Å × 17.38 Å × 10.96 Å.

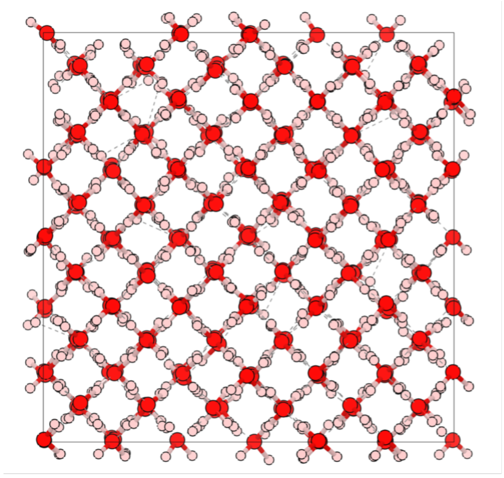

Figure S4: **The structure of ice VII used in the production of the ice VII data points in Fig. 3 in the main paper.** In this structure, oxygens are shown in red and hydrogens in pink.

This system is also the origin of the pressurized water systems. When ice VII is simulated at a temperature of 500 K and a pressure below 9.5 GPa, the system melts. The calculations for Fig. 3B are performed in the NPT ensemble with an anisotropic thermostat. The calculations for Fig. 3D are performed in the NVT ensemble in a cell with the average NPT volume; this cell has dimensions 24.39 Å × 24.38 Å × 15.37 Å.

S1.4: Superionic silver iodide

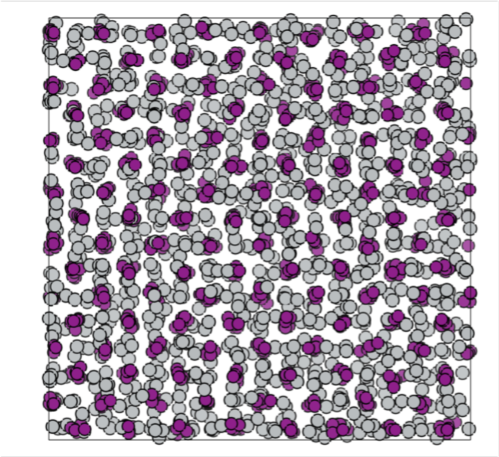

Figure S5: **Structure of the silver iodide system used for the analyses in this supporting document.** Silver is shown in silver and iodine in purple. This structure is an 8×8×8 expansion of the primitive cell.

Simulations of silver iodide in section S3 were performed using the machine-learned MACE model (*75*) trained on a set of PBE-D3 DFT calculations previously developed and validated by Hajibabaei *et al.* (*76*) Simulations were performed using ASE in the NVT ensemble, with the volume set to match the equilibrium volume obtained from an NPT simulation. A Nosé-Hoover thermostat with a relaxation time of 1 ps was applied. A timestep of 1 fs was used due to the high mass of silver, and each simulation was run for 1 ns.

The structure of superionic silver iodide—shown in Fig. S5—consists of 2048 atoms in an 8 × 8 × 8 expansion of a primitive cell (40.62 Å × 40.62 Å × 40.62 Å). For each temperature, the volume of the cell is taken from the equilibrium volume of a simulation run with a Nosé-Hoover barostat at a pressure of 1 bar. A smaller cell—a 4 × 4 × 4 expansion of the primitive, containing

256 atoms—generated in the same way was used for the ICOBI calculations below.

S2: Details of simulations and sampling used to make figures in the main text and in this supporting information

The actual dynamical simulations from which the analyses in the main paper and of silver iodide in section S3 are calculated are listed in Table S1.

Table S1: Summary of simulations and corresponding figures derived from them.

System	Temp.	Len.	Atoms	Figs. Calc.	Figs. Deriv.
Nanoconfined superionic water (12 GPa)	500 K	500 ps	432	1B, C; 2A, B, C; 3	1D (10 structures)
Nanoconfined water from Ref. 2	500 K[a]	1 ns	432	3B	–
Nanoconfined superionic water (12 GPa)	425–550 K (25 K steps)	500 ps	432	S7	–
Bulk superionic water (BCC and FCC)	2500 K	1 ns	384	1A, C, S12 (FCC)	1D (10 structures)
Ice VII (50000 GPa)	500 K	2 ns	864	3C	–
Pressurised water (500 GPa)	500 K	2 ns	864	3D	–
Simulations of pressurised water and Ice VII in the NPT ensemble	500 K	500 ps	864	3B	–
Superionic AgI from Ref. 4	480 K	1 ns	2048	S5, S6	–

S3: Structure, bonding, and chain like diffusion in superionic silver iodide

In the main paper in Fig. 1, we present structure and bonding calculations for bulk and nanoconfined superionic water and note the similarities and differences between the two systems. In Fig. S6, we

present the same information for superionic silver iodide. In general, we can observe similarities with the structure of BCC-ordered bulk superionic water. The snapshot in A and the form of the RDF in B are exceptionally similar, but a change in distances in the RDFs arises due to the far greater lattice constant of AgI.

We should, however, note that the form of the ICOBI plot is somewhat different from that of bulk superionic water. Though we see a similar distribution, it is centred at a similar level of ionic interactions. This difference can be attributed to two factors: firstly, the greater electronegativity gap between silver and iodine will lead to greater ionicity in bonding (*29*); and secondly, unlike hydrogen, silver is capable of having a far higher coordination number than two (the optimal value for a proton), leading to a greater number of weaker covalent interactions being formed with neighboring iodide ions (*32*).

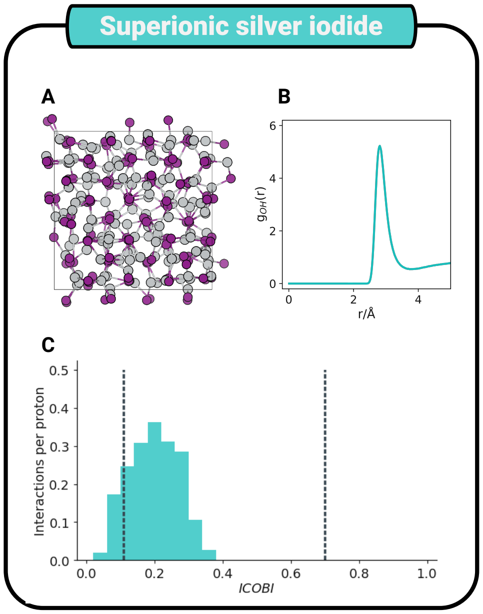

Figure S6: **A companion figure to Fig. 1 in the main paper for superionic silver iodide.** It comprises, (A) the structure of the phase, (B) the $g_{\mathrm{AgI}}(r)$ 480 K, and (C) a plot of the ICOBI index for individual Ag–I for a snapshot taken at 480 K.

In addition to similarities in structure with bulk superionic water, we also observe chain-like diffusion in superionic silver iodide. In Fig. S7, we investigate AgI using the same analysis as for the two superionic water-based systems in Fig. 2 of the main text, with δ = 1.75 Å and Δt = 1 ps.

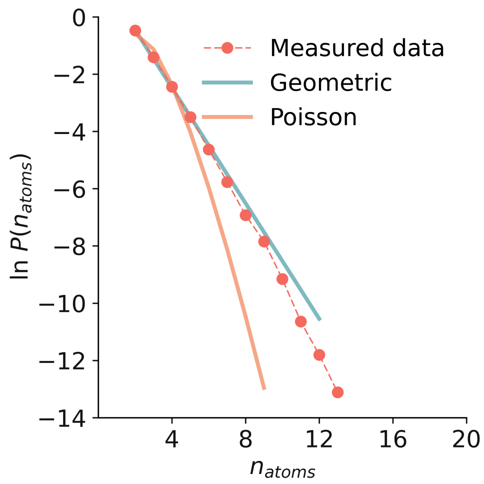

Figure S7: **Plot of the log probability of chain lengths for silver iodide at 480 K.** Geometric and Poisson fits to the data are shown, with the greater observed agreement with the geometric law indicating a chain-like diffusion mechanism. The parameters in this plot are taken as δ = 1.75 Å and Δt = 1 ps.

As with both forms of superionic water studied in the main paper, we observe better agreement with the geometric model. This is indicative of a chain-like diffusion mechanism.

S4: Calculation of pKw from equilibrium simulations of nanoconfined superionic water

The calculation of pK_w from molecular simulations usually requires the employment of some form of advanced sampling technique. In nanoconfined superionic water, the level of ionisation is so high that the pK_w can be calculated directly from the concentration of defect ions, using the Voronoi tessellation method for identifying defects. If we assume that the activity of pure nanoconfined water is

$$pK_w = -\log_{10}\left([\mathrm{OH}^-]\left[\mathrm{H_3O}^+\right]\right). \quad \text{(S1)}$$

In Fig. S8 we report these results for nanoconfined superionic water from 420 K to 550 K. We observe values ranging from just above 3 to 1.8. These values are vastly different from the values

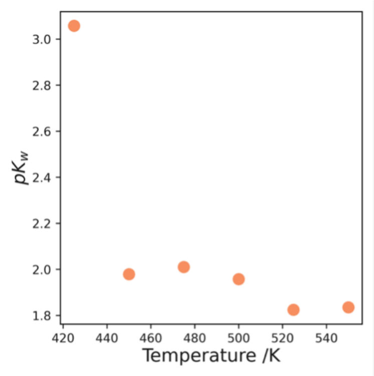

Figure S8: **Plots of pK_w calculated with temperature for nanoconfined superionic water**. These values are based on defect populations obtained from Voronoi tessellation.

previously obtained for nanoconfined water at atmospheric pressure using advanced sampling techniques (*77, 78*).

S5: The effect of the inclusion of nuclear quantum effects on the structure of nanoconfined superionic water

In this paper, we have focused on the qualitative description of the two types of superionic. While these two phases have been shown to be qualitatively different, it may be reasonably asked whether there is a profound change in the structure of nanoconfined superionic water when nuclear quantum effects (NQEs) are introduced. To these ends, we use simulations produced by Ravindra *et al.* (*19*) and compare the radial distribution function and the distribution of water bond angle with and without the inclusion of nuclear quantum effects (with the position of protons in the NQE inclusive plot taken as the positon of centroids). We observe in these plots shown in Fig. S9 that the inclusion of nuclear quantum effects, does not change the distributions beyond the expected broadening of peaks in both distributions. As a quantum hydrogen nuclei would be expected to be more diffuse in the bulk superionic phase as well, we can conclude that the qualitative differences described in this paper are not affected by quantum descriptions of nuclear motion.

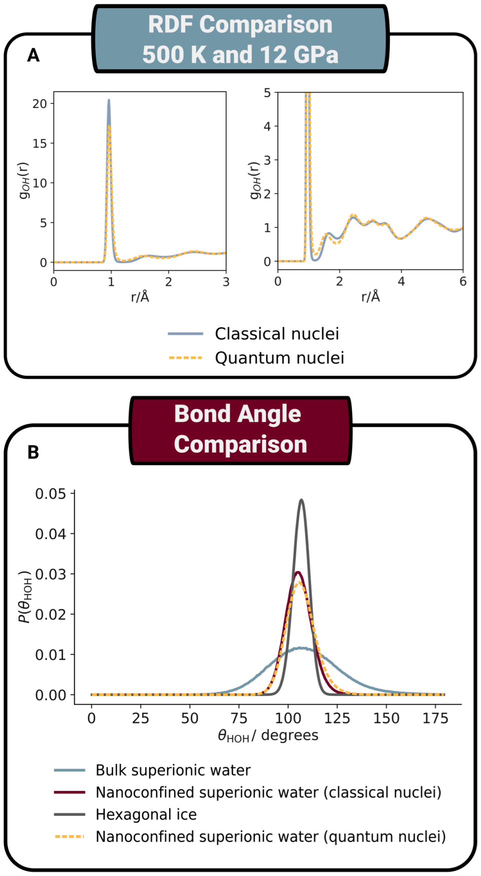

Figure S9: **Plots exploring the effects of a quantum nuclear description on the structural descriptors presented for nanoconfined superionic water in the main paper.** Panel (A) shows the effect of this description on the oxygen-hydrogen radial distribution function. To allow for the full appraisal of the the effect in structure a zoom of the full distribution is shown on the right hand side. Panel (B) reproduces the plot of the bond angle in the main paper with the values for quantum nuclei in nanoconfined superionic water added. In the NQE inclusive plots we plot the location of centroids as a proxy for the location of hydrogen nuclei.

S6: Applying Voronoi tessellation to locate defects in nanoconfined superionic water

The difference in the defect chemistry of nanoconfined and bulk superionic water becomes apparent if we try to allocate bulk defects as we do in the nanoconfined system, as shown in Fig. S10. In the nanoconfined system, defects are allocated by Voronoi tessellation. When we do so, we identify that most oxygens have a coordination number of 2, with the small number of coordination numbers 1 and 3 representing the defects.

If we do this on the bulk system, we obtain a broad distribution of coordination numbers, with 0 and 4 both widely observed. This is not demonstrative of a high number of defects, but rather that the nature of defects is fundamentally different, with bulk superionic water hydrogen defects being the conventional crystalline vacancies and interstitials. These are the conductive defects, instead of hydroxyl and hydronium ions.

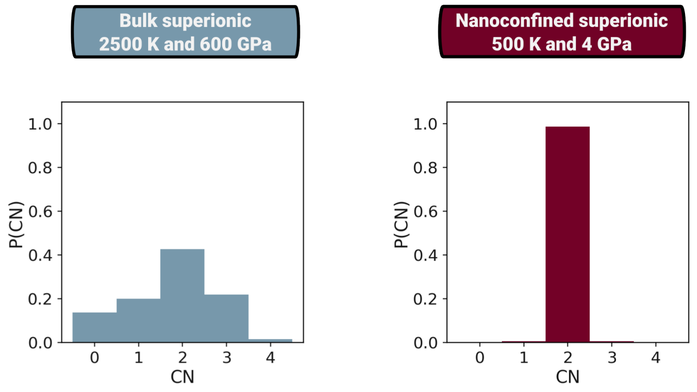

Figure S10: **Plots of coordination numbers in the bulk and nanoconfined system obtained by Voronoi tessellation.** Shown for bulk superionic water (2500 K, 600 GPa) and nanoconfined superionic water (600 K, 12 GPa).

S7: Comparison of bulk superionic water neural network potential with hybrid DFT calculations

In the main paper, we have compared and contrasted the structures and diffusive mechanisms of bulk superionic water and nanoconfined superionic water. In order to accurately simulate nanoconfined superionic water, Kapil et al. used a hybrid functional to fit the neural network potential (*15*). However, the bulk superionic potential used by Cheng (*12, 59*) and co-workers was fit from revPBE-D3 level DFT.

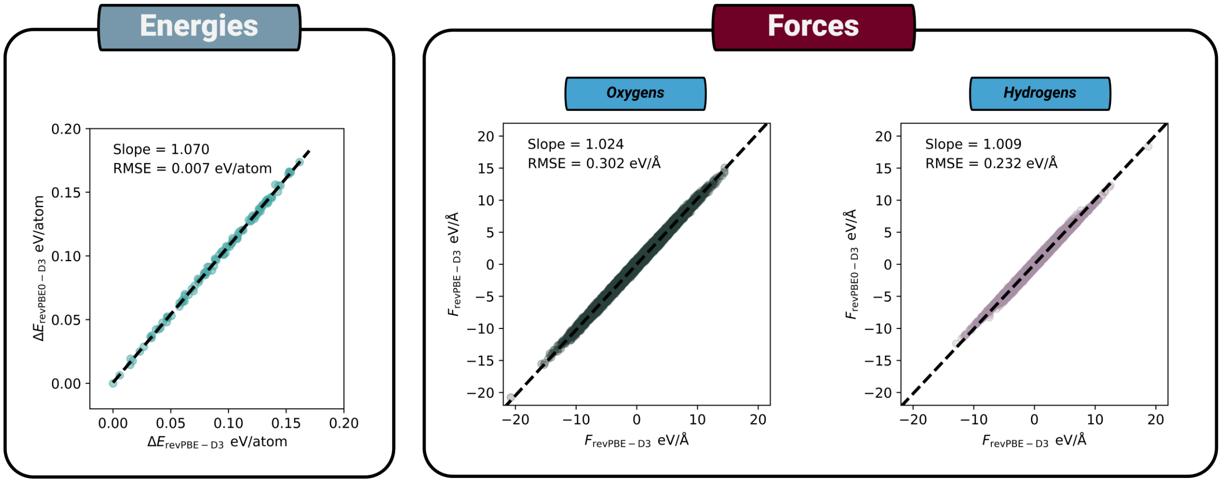

Figure S11: **Plots of the energy and atomic forces of frames obtained with the bulk revPBE-D3 compared to revPBE-D3 calculations.**

Though this potential was properly validated, it is worth considering if we would observe qualitatively different results if the potential had been fit with a hybrid DFT functional. To consider this, we have taken structures from a trajectory of bulk superionic water at 2500 K and 600 GPa. Structures were selected from the trajectory by ranking structures by energy and taking 100 structures at equal increments along this energy scale.

As the model was fit using plane wave DFT calculations the energies of the two models cannot be directly compared. To rectify for this we perform the comparison in energies and forces from Density functional theory calculations were performed using the CP2K code at the revPBE level (the same as was used to fit the model) and revPBE0-D3 level of theory (used for the nanoconfined water model) for all other settings those used to fit the model by Kapil *et al.* (*15*) are used for both

sets of calculations. We plot the resulting energies of frames against each other in Fig. S11, where we see a good agreement in the energetic ordering of structures between functionals; though, of course, the RMSE errors are substantially greater than those obtained during a fitting process.

Further, the slope of the line of best fit is close to one; this suggests the overall width of the configurational density of states relative to the thermal energy is similar for systems with interactions described by the two functionals. This suggests that the physics described by the neural network potential should be qualitatively similar to that we would obtain at a hybrid level of theory.

We further observe similar agreement for forces acting on hydrogens and oxygens in these frames. This suggests that the potential energy surfaces described at these two levels of theory are similar enough in form and lend force to the qualitative conclusions we have drawn in this paper.

S8: FCC structural information

In Fig. S12, fcc superionic water is analyzed using the same structural criteria applied to the bcc phase in the main text. The left panel shows the bond-angle distribution for fcc superionic water, which is broader than that observed in molecular water-based systems and is similar in character to that of the bcc superionic phase. While fcc and bcc superionic water share common features, their bond-angle distributions differ in detail, reflecting differences in the underlying lattice symmetries. In particular, the lower peak height in the fcc distribution is compensated by an extended tail at larger angles, which is not easily resolved on the present scale. Moreover, although a residual local angular structure is present, the radial distribution function shown in Fig. S12 is characteristic of inorganic crystalline systems and lacks the sharp, high-amplitude peaks associated with well-defined molecular bonding, in contrast to the nanoconfined molecular superionic phase.

The string analysis shown in the rightmost panel of Fig. S12 exhibits the same geometric distribution of chain lengths observed for the bulk superionic conductors discussed in the main text.

Together, these results demonstrate that the criteria used to identify bulk superionic behavior apply consistently across both superionic phases of bulk water, thereby reinforcing the conclusions of the main manuscript.

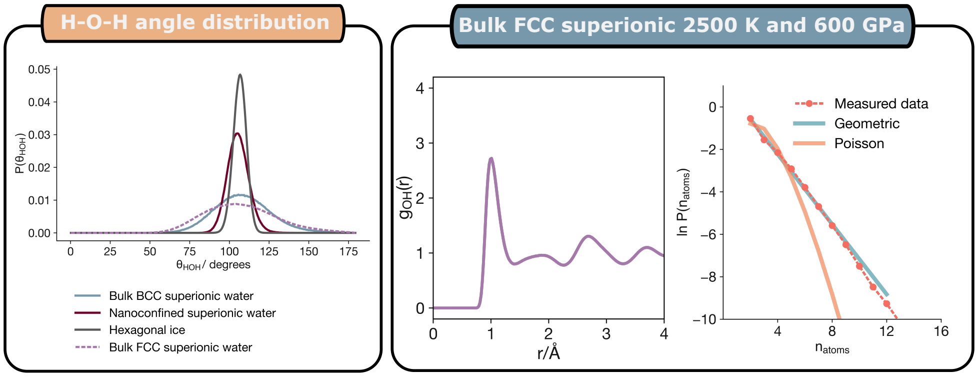

Figure S12: **Structural and dynamical characterization of fcc-ordered bulk superionic water using the same criteria applied to bcc superionic water in the main text.** On the left, H-O-H bond-angle distributions are compared with those of the three ice phases discussed in the main paper. In the center, the O–H radial distribution function is shown for fcc bulk superionic water at 2500 K and 600 GPa. On the right, a string-length analysis characterizing collective proton transport is presented, computed with $\Delta t = 0.2$ ps and $\delta = 1.5$ Å.

S9: Calculation of the conductivity of nanoconfined superionic water

The ionic conductivity of nanoconfined superionic water at 12 GPa and 500 K was calculated using the approach developed by French *et al.* (*79*) previously in detail for the same system by Ravindra *et al.* (*19*). In this approach, the conductivity is estimated by defining lower and upper bounds based on the diffusion coefficients of hydrogen and oxygen. The lower bound is given by

$$\sigma_{\text{lower}} = \frac{\rho e^2 \left(D_H - D_O \right)}{k_B T}, \tag{S2}$$

and the upper bound by

$$\sigma_{\text{upper}} = \frac{\rho e^2 D_H}{k_B T}. \tag{S3}$$

Here, ρ denotes the proton number density, e the elementary charge, k_B the Boltzmann constant, and T the temperature, while D_H and D_O are the diffusion coefficients of hydrogen and oxygen, respectively.

The diffusion coefficients were obtained from the mean-squared displacements shown in Fig. S13 using the kinisi software (*68, 69*). Applying the expressions above yields lower and

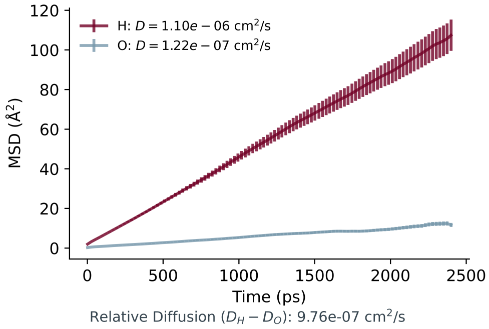

Figure S13: **Plots of mean squared displacements for hydrogen and oxygen in nanoconfined superionic water at 12 GPa and 400 K.** Errors are calculated using the method described by McCluskey *et al.* (*68*).

upper bounds for the ionic conductivity of 0.15 S/cm and 0.17 S/cm, respectively. These values fall within the confidence interval of the conductivity reported by Ravindra *et al.* (*19*), providing consistency with prior quantitative estimates.

S10: Sensitivity analysis of the parameters for calculating chains

In this paper, we have considered the possibility of concerted diffusion mechanisms from the perspective of the chain-like diffusion descriptors previously used to describe diffusion in glasses (*72*) and solid electrolytes (*43*). Chains are identified on a pairwise basis, where one atom—in our case limited to the hydrogen atoms in the system—has moved away from its initial site and been replaced by another. This is defined mathematically for atoms with positions $\mathbf{r}_i$ and $\mathbf{r}_j$ at time t as,

$$\min\left[\left\|r_{\mathrm{i}}(t+\Delta t)-r_j(t)\right\|,\left\|r_j(t+\Delta t)-r_j(t)\right\|\right]<\delta \tag{S4}$$

For a time window Δt and a spatial cutoff δ, the value of these variables needs to be set and has a quantitative effect on the length of chains. However, the qualitative results obtained have previously been suggested to have limited dependence on these parameters in Lennard-Jones glasses, provided that the spatial cutoff is smaller than the hard sphere radius. In crystalline solid electrolytes, this cutoff can be meaningfully set based on the periodicity of the framework lattice. Given the two-step diffusion mechanism of nanoconfined superionic water, the exact setting of the spatial cutoff is

difficult, and definitions based on both the excluded volume of protons and water molecules can be argued for. To assess whether the qualitative conclusions in the main paper are robust to the choice of these values, we performed a sensitivity analysis. Regardless of the chosen values, we observe a geometric decay in probability in chain lengths for both nanoconfined and bulk superionic water, giving a strong degree of confidence in both cases that diffusion is chain-like. Plots are shown below for both varieties of superionic water for spatial cutoffs of 1 Å, 1.5 Å, and 2 Å for multiple time windows for each system.

Nanoconfined superionic water

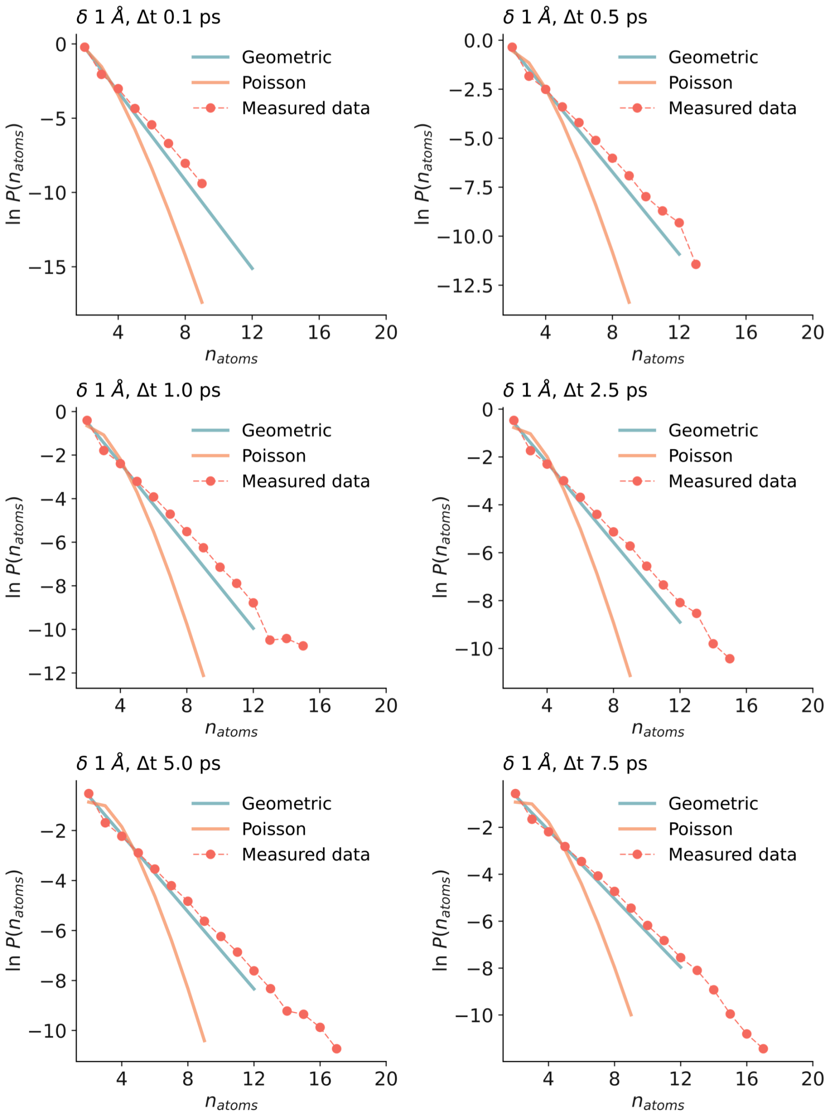

Figure S14: **Plots of the log probability of the lengths of diffusive chains for nanoconfined superionic water with a spatial cutoff (δ) of 1 Å, and time windows of Δt of: 0.1 ps, 0.5 ps, 1.0 ps, 2.5 ps, 5.0 ps, and 7.5 ps.** Fits to Geometric and Poisson models are provided; these models are representative of chain-like and isolated hopping-based diffusion.

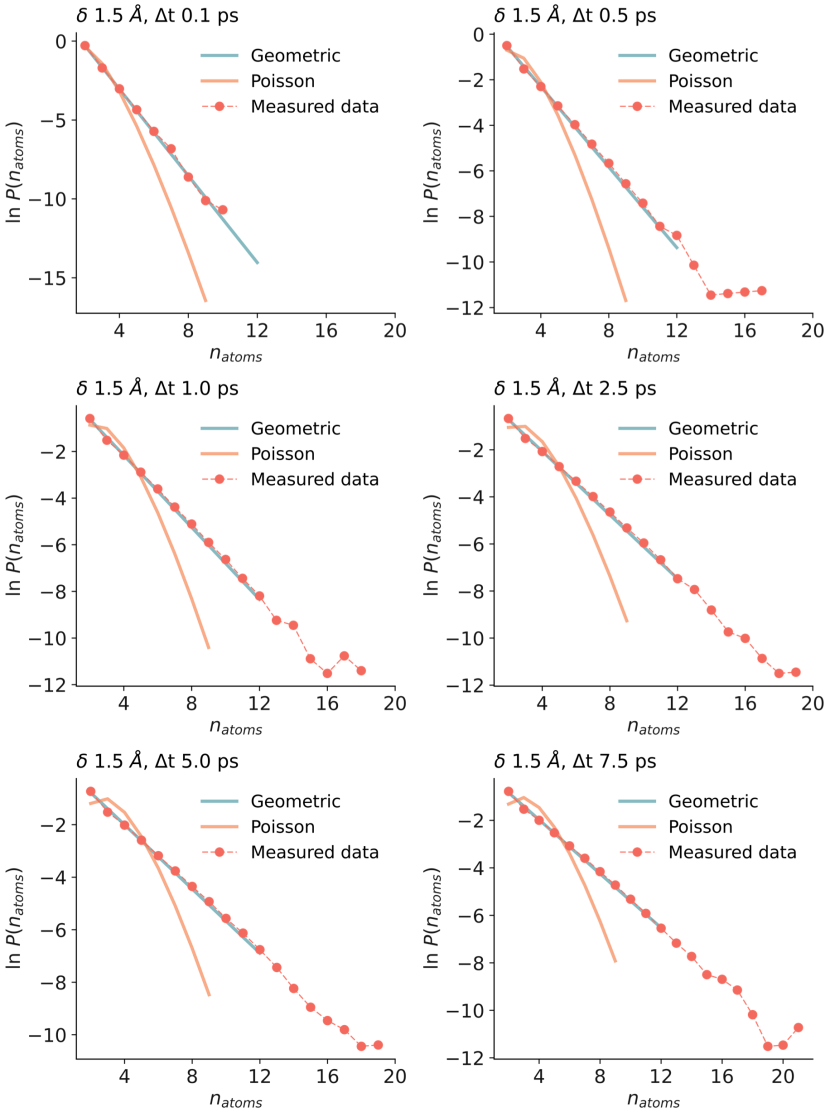

Figure S15: **Plots of the log probability of the lengths of diffusive chains for nanoconfined superionic water with a spatial cutoff (δ) of 1.5 Å, and time windows of Δt of: 0.1 ps, 0.5 ps, 1.0 ps, 2.5 ps, 5.0 ps, and 7.5 ps.** Fits to Geometric and Poisson models are provided; these models are representative of chain-like and isolated hopping-based diffusion.

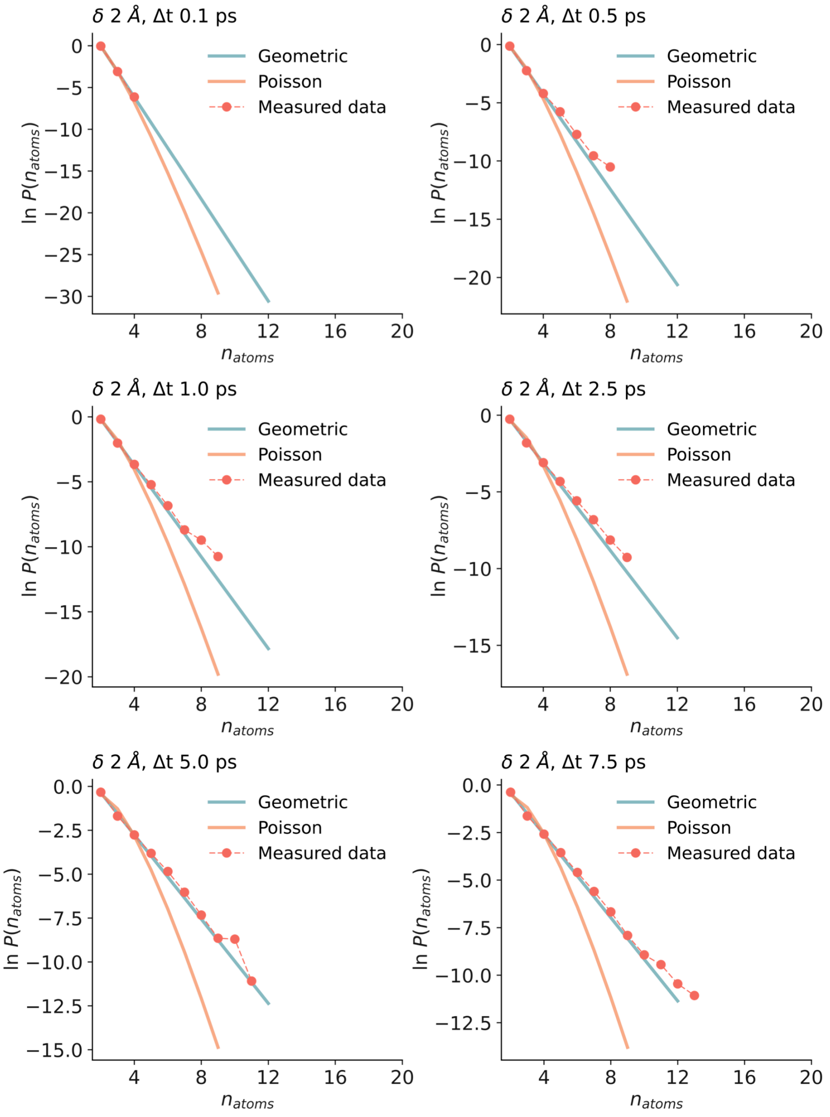

Figure S16: **Plots of the log probability of the lengths of diffusive chains for nanoconfined superionic water with a spatial cutoff (δ) of 2 Å, and time windows of Δt of: 0.1 ps, 0.5 ps, 1.0 ps, 2.5 ps, 5.0 ps, and 7.5 ps.** Fits to Geometric and Poisson models are provided; these models are representative of chain-like and isolated hopping-based diffusion.

Bulk superionic water

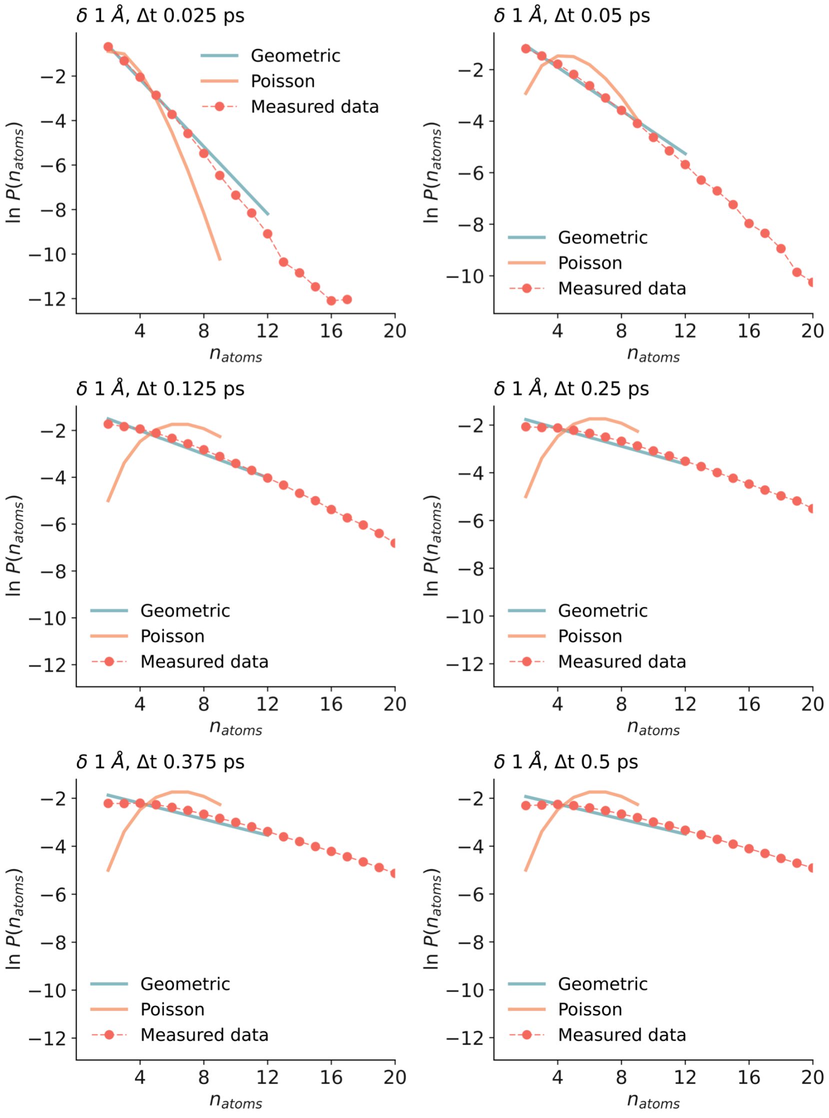

Figure S17: **Plots of the log probability of the lengths of diffusive chains for bulk superionic water with a spatial cutoff (δ) of 1 Å, and time windows of Δt of: 0.025 ps, 0.05 ps, 0.125 ps, 0.25 ps, 0.375 ps, and 0.5 ps.** Fits to Geometric and Poisson models are provided; these models are representative of chain-like and isolated hopping-based diffusion.

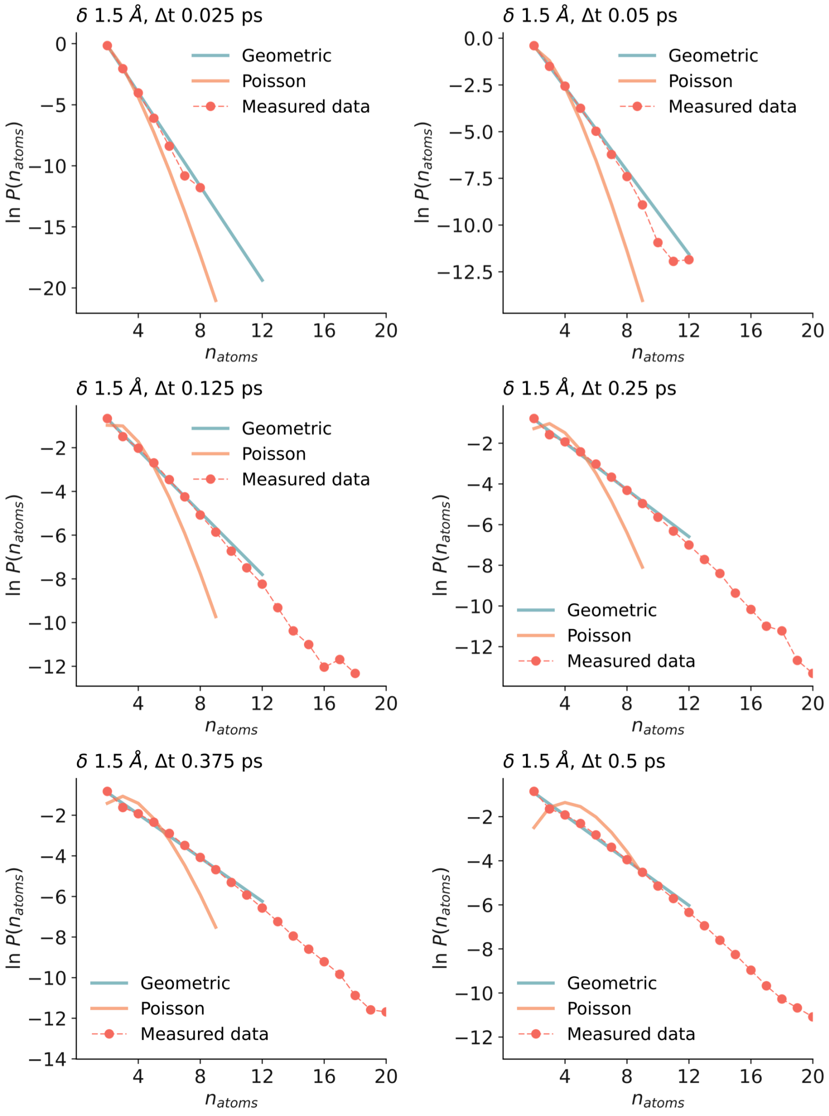

Figure S18: **Plots of the log probability of the lengths of diffusive chains for bulk superionic water with a spatial cutoff (δ) of 1.5 Å, and time windows of Δt of: 0.025 ps, 0.05 ps, 0.125 ps, 0.25 ps, 0.375 ps, and 0.5 ps.** Fits to Geometric and Poisson models are provided; these models are representative of chain-like and isolated hopping-based diffusion.

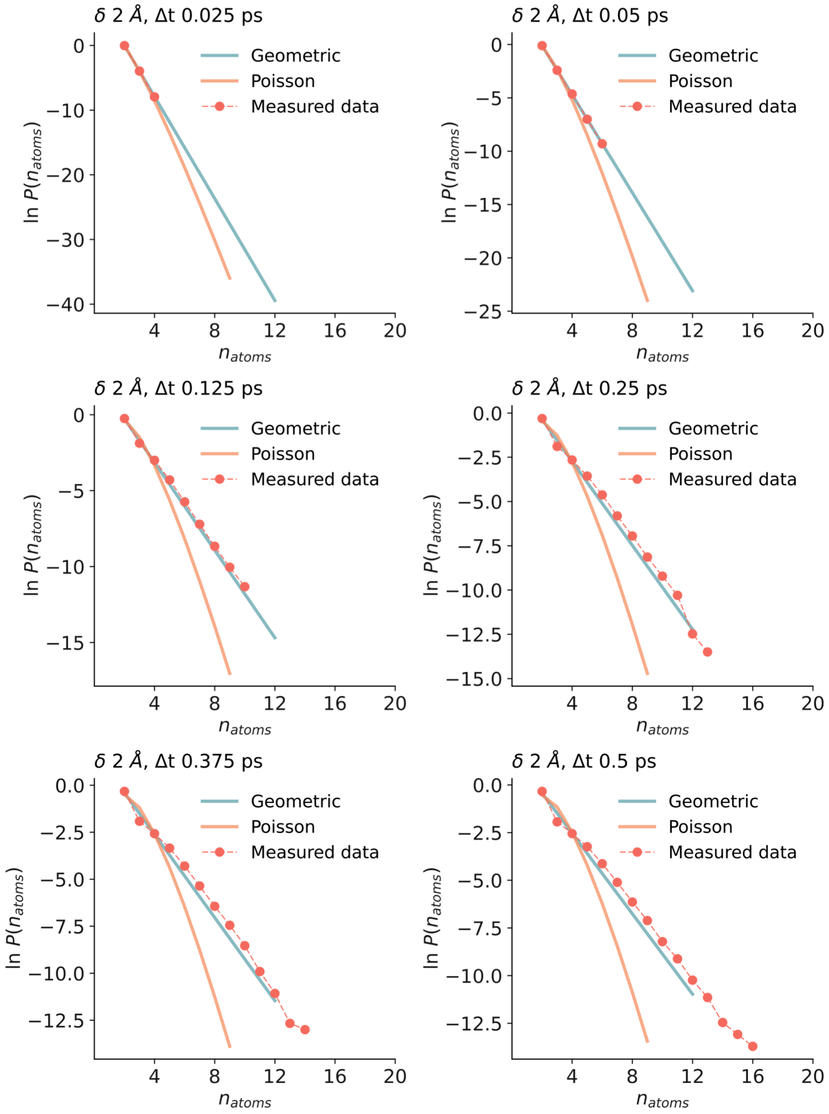

Figure S19: **Plots of the log probability of the lengths of diffusive chains for bulk superionic water with a spatial cutoff (δ) of 2 Å, and time windows of Δt of: 0.025 ps, 0.05 ps, 0.125 ps, 0.25 ps, 0.375 ps, and 0.5 ps.** Fits to Geometric and Poisson models are provided; these models are representative of chain-like and isolated hopping-based diffusion.

Supplementary movies

To support this manuscript two supplementary movies featuring the diffusion of hydroxide and hydronium defects are provided are available on the journal website. The exact content of these movies is descibed below:

Supplementary Movie 1 A video showing a molecular dynamics trajectory of nanoconfined superionic with defects indicated. The hydronium defect is shown in (yellow), and the hydroxide in (green), the periodic boundary is shown as a hashed line.

Supplementary Movie 2 A video showing a molecular dynamics trajectory of nanoconfined superionic water with the motion of defects tracked. The hydronium defect is shown in (yellow), and the hydroxide in (green), with trails of the same colour showing their path through the trajectory. The periodic boundary is shown as black hashed line, only the defect which initially fell within the boundary is tracked.

REFERENCES

1. K. Funke, Solid state ionics: From Michael Faraday to green energy–The European dimension. *Sci. Technol. Adv. Mater.* **14**, 043502 (2013).

2. M. Faraday, On the magnetization of light and the illumination of magnetic lines of force. *Philos. Trans. R. Soc. Lond.* **4**, 49–52 (1843).

3. M. Rice, W. Roth, Ionic transport in super ionic conductors: A theoretical model. *J. Solid State Chem.* **4**, 294–310 (1972).

4. V. Thangadurai, H. Kaack, W. J. F. Weppner, Novel fast lithium ion conduction in garnet-type $Li_5La_3M_2O_{12}$ (M = Nb, Ta). *J. Am. Ceram. Soc.* **86**, 437–440 (2003).

5. R. Murugan, V. Thangadurai, W. Weppner, Fast lithium ion conduction in garnet-type $Li_7La_3Zr_2O_{12}$. *Angew. Chem. Int. Ed. Engl.* **46**, 7778–7781 (2007).

6. N. Kamaya, K. Homma, Y. Yamakawa, M. Hirayama, R. Kanno, M. Yonemura, T. Kamiyama, Y. Kato, S. Hama, K. Kawamoto, A. Mitsui, A lithium superionic conductor. *Nat. Mater.* **10**, 682–686 (2011).

7. B. A. Paren, N. Nguyen, V. Ballance, D. T. Hallinan, J. G. Kennemur, K. I. Winey, Superionic Li-ion transport in a single-ion conducting polymer blend electrolyte. *Macromolecules* **55**, 4692–4702 (2022).

8. Y. Wang, A. P. Sokolov, Design of superionic polymer electrolytes. *Curr. Opin. Chem. Eng.* **7**, 113–119 (2015).

9. M. Millot, S. Hamel, J. R. Rygg, P. M. Celliers, G. W. Collins, F. Coppari, D. E. Fratanduono, R. Jeanloz, D. C. Swift, J. H. Eggert, Experimental evidence for superionic water ice using shock compression. *Nat. Phys.* **14**, 297–302 (2018).

10. P. Demontis, R. LeSar, M. L. Klein, New high-pressure phases of ice. *Phys. Rev. Lett.* **60**, 2284–2287 (1988).

11. V. B. Prakapenka, N. Holtgrewe, S. S. Lobanov, A. F. Goncharov, Structure and properties of two superionic ice phases. *Nat. Phys.* **17**, 1233–1238 (2021).

12. B. Cheng, M. Bethkenhagen, C. J. Pickard, S. Hamel, Phase behaviours of superionic water at planetary conditions. *Nat. Phys.* **17**, 1228–1232 (2021).

13. N. Goldman, L. E. Fried, I.-F. W. Kuo, C. J. Mundy, Bonding in the superionic phase of water. *Phys. Rev. Lett.* **94**, 217801 (2005).

14. F. Matusalem, J. Santos Rego, M. de Koning, Plastic deformation of superionic water ices. *Proc. Natl. Acad. Sci. U.S.A.* **119**, e2203397119 (2022).

15. V. Kapil, C. Schran, A. Zen, J. Chen, C. J. Pickard, A. Michaelides, The first-principles phase diagram of monolayer nanoconfined water. *Nature* **609**, 512–516 (2022).

16. G. Algara-Siller, O. Lehtinen, F. C. Wang, R. R. Nair, U. Kaiser, H. A. Wu, A. K. Geim, I. V. Grigorieva, Square ice in graphene nanocapillaries. *Nature* **519**, 443–445 (2015).

17. R. Wang, M. Souilamas, A. Esfandiar, R. Fabregas, S. Benaglia, H. Nevison-Andrews, Q. Yang, J. Normansell, P. Ares, G. Ferrari, A. Principi, A. K. Geim, L. Fumagalli, In-plane dielectric constant and conductivity of confined water. arXiv:2407.21538 [cond-mat.mes-hall] (2025).

18. J. Jiang, Y. Gao, L. Li, Y. Liu, W. Zhu, C. Zhu, J. S. Francisco, X. C. Zeng, Rich proton dynamics and phase behaviours of nanoconfined ices. *Nat. Phys.* **20**, 456–464 (2024).

19. P. Ravindra, X. R. Advincula, B. X. Shi, S. W. Coles, A. Michaelides, V. Kapil, Nuclear quantum effects induce superionic proton transport in nanoconfined water. arXiv:2410.03272 [cond-mat.mtrl-sci] (2024).

20. D. Laage, J. T. Hynes, A molecular jump mechanism of water reorientation. *Science* **311**, 832–835 (2006).

21. A. Hassanali, F. Giberti, J. Cuny, T. D. Kühne, M. Parrinello, Proton transfer through the water gossamer. *Proc. Natl. Acad. Sci. U.S.A.* **110**, 13723–13728 (2013).

22. J. D. Bernal, R. H. Fowler, A theory of water and ionic solution, with particular reference to hydrogen and hydroxyl ions. *J. Chem. Phys.* **1**, 515–548 (1933).

23. B. Das, S. Ruiz-Barragan, B. Bagchi, D. Marx, Topological frustration triggers ultrafast dynamics of monolayer water confined in graphene slit pores. *Nano Lett.* **24**, 15623–15628 (2024).

24. J. Sun, B. K. Clark, S. Torquato, R. Car, The phase diagram of high-pressure superionic ice. *Nat. Commun.* **6**, 8156 (2015).

25. Y. Wang, W. D. Richards, S. P. Ong, L. J. Miara, J. C. Kim, Y. Mo, G. Ceder, Design principles for solid-state lithium superionic conductors. *Nat. Mater.* **14**, 1026–1031 (2015).

26. B. Morgan, P. A. Madden, Ion mobilities and microscopic dynamics in liquid (Li,K)Cl. *J. Chem. Phys.* **120**, 1402–1413 (2004).

27. M. Simoes Santos, M. Salanne, T. Kooyman, D. Lambertin, Structural and transport properties of the molten salt NaCl-KCl-UCl3 using the polarizable ion model. *J. Nucl. Mater.* **597**, 155125 (2024).

28. S. Hull, Superionics: Crystal structures and conduction processes. *Rep. Prog. Phys.* **67**, 1233–1314 (2004).

29. P. C. Müller, C. Ertural, J. Hempelmann, R. Dronskowski, Crystal orbital bond index: Covalent bond orders in solids. *J. Phys. Chem. C* **125**, 7959–7970 (2021).

30. S. Ninet, F. Datchi, A. M. Saitta, Proton disorder and superionicity in hot dense ammonia ice. *Phys. Rev. Lett.* **108**, 165702 (2012).

31. C. J. Pickard, R. J. Needs, Highly compressed ammonia forms an ionic crystal. *Nat. Mater.* **7**, 775–779 (2008).

32. K.-D. Kreuer, Proton conductivity: Materials and applications. *Chem. Mater.* **8**, 610–641 (1996).

33. B. C. Wood, J. B. Varley, K. E. Kweon, P. Shea, A. T. Hall, A. Grieder, M. Ward, V. P. Aguirre, D. Rigling, E. Lopez Ventura, C. Stancill, N. Adelstein, Paradigms of frustration in superionic solid electrolytes. *Philos. Trans. R. Soc A Math. Phys. Eng. Sci.* **379**, 20190467 (2021).

34. C. R. A. Catlow, Atomistic mechanisms of ionic transport in fast-ion conductors. *J. Chem. Soc. Faraday Trans.* **86**, 1167 (1990).

35. B. J. Morgan, P. A. Madden, Relationships between atomic diffusion mechanisms and ensemble transport coefficients in crystalline polymorphs. *Phys. Rev. Lett.* **112**, 145901 (2014).

36. M. Joos, X. Kang, R. Merkle, J. Maier, Water uptake of solids and its impact on ion transport. *Nat. Mater.* **24**, 397–403 (2025).

37. C. J. D. de Grotthuss, *Mémoire sur la décomposition de l'eau: et des corps qu'elle tient en dissolution à l'aide de l'électricité galvanique* (Rome) (1805); https://books.google.com/books?id=ORxIjwEACAAJ.

38. D. Marx, Proton transfer 200 years after von Grotthuss: Insights from ab initio simulations. *ChemPhysChem* **7**, 1848–1870 (2006).

39. M. Chen, L. Zheng, B. Santra, H.-Y. Ko, R. A. DiStasio Jr., M. L. Klein, R. Car, X. Wu, Hydroxide diffuses slower than hydronium in water because its solvated structure inhibits correlated proton transfer. *Nat. Chem.* **10**, 413–419 (2018).

40. M. E. Tuckerman, A. Chandra, D. Marx, Structure and dynamics of OH-(aq). *Acc. Chem. Res.* **39**, 151–158 (2006).

41. D. Marx, M. E. Tuckerman, J. Hutter, M. Parrinello, The nature of the hydrated excess proton in water. *Nature* **397**, 601–604 (1999).

42. M. Burbano, D. Carlier, F. Boucher, B. J. Morgan, M. Salanne, Sparse cyclic excitations explain the low ionic conductivity of stoichiometric $Li_7La_3Zr_2O_{12}$. *Phys. Rev. Lett.* **116**, 135901 (2016).

43. B. J. Morgan, Mechanistic origin of superionic lithium diffusion in anion-disordered Li_6PS_5X argyrodites. *Chem. Mater.* **33**, 2004–2018 (2021).

44. A. Annamareddy, J. Eapen, Low dimensional string-like relaxation underpins superionic conduction in fluorites and related structures. *Sci. Rep.* **7**, 44149 (2017).

45. Z. Futera, J. S. Tse, N. J. English, Possibility of realizing superionic ice VII in external electric fields of planetary bodies. *Sci. Adv.* **6**, eaaz2915 (2020).

46. N. Noguchi, T. Okuchi, Self-diffusion of protons in H_2O ice VII at high pressures: Anomaly around 10 GPa. *J. Chem. Phys.* **144**, 234507 (2016).

47. X.-Z. Li, M. I. J. Probert, A. Alavi, A. Michaelides, Quantum nature of the proton in water-hydroxyl overlayers on metal surfaces. *Phys. Rev. Lett.* **104**, 066102 (2010).

48. A. Gomez, W. H. Thompson, D. Laage, Neural-network-based molecular dynamics simulations reveal that proton transport in water is doubly gated by sequential hydrogen-bond exchange. *Nat. Chem.* **16**, 1838–1844 (2024).

49. A. Chandra, M. E. Tuckerman, D. Marx, Connecting solvation shell structure to proton transport kinetics in hydrogen-bonded networks via population correlation functions. *Phys. Rev. Lett.* **99**, 145901 (2007).

50. P. Ravindra, X. R. Advincula, C. Schran, A. Michaelides, V. Kapil, Quasi-one-dimensional hydrogen bonding in nanoconfined ice. *Nat. Commun.* **15**, 7301 (2024).

51. G. Tocci, A. Michaelides, Solvent-induced proton hopping at a water-oxide interface. *J. Phys. Chem. Lett.* **5**, 474–480 (2014).

52. C. Schran, F. L. Thiemann, P. Rowe, E. A. Müller, O. Marsalek, A. Michaelides, Machine learning potentials for complex aqueous systems made simple. *Proc. Natl. Acad. Sci. U.S.A.* **118**, e2110077118 (2021).

53. F. Della Pia, A. Zen, D. Alfè, A. Michaelides, DMC-ICE13: Ambient and high pressure polymorphs of ice from diffusion Monte Carlo and density functional theory. *J. Chem. Phys.* **157**, 134701 (2022).

54. J. G. Brandenburg, A. Zen, M. Fitzner, B. Ramberger, G. Kresse, T. Tsatsoulis, A. Grüneis, A. Michaelides, D. Alfè, Physisorption of water on graphene: Subchemical accuracy from many-body electronic structure methods. *J. Phys. Chem. Lett.* **10**, 358–368 (2019).

55. V. Kapil, M. Rossi, O. Marsalek, R. Petraglia, Y. Litman, T. Spura, B. Cheng, A. Cuzzocrea, R. H. Meißner, D. M. Wilkins, B. A. Helfrecht, P. Juda, S. P. Bienvenue, W. Fang, J. Kessler, I. Poltavsky, S. Vandenbrande, J. Wieme, C. Corminboeuf, T. D. Kühne, D. E. Manolopoulos, T. E. Markland, J. O. Richardson, A. Tkatchenko, G. A. Tribello, V. Van Speybroeck, M. Ceriotti, i-PI 2.0: A universal force engine for advanced molecular simulations. *Comput. Phys. Commun.* **236**, 214–223 (2019).

56. A. Singraber, J. Behler, C. Dellago, Library-based LAMMPS implementation of high-dimensional neural network potentials. *J. Chem. Theory Comput.* **15**, 1827–1840 (2019).

57. A. Hjorth Larsen, J. Jørgen Mortensen, J. Blomqvist, I. E. Castelli, R. Christensen, M. Dułak, J. Friis, M. N. Groves, B. Hammer, C. Hargus, E. D. Hermes, P. C. Jennings, P. Bjerre Jensen, J. Kermode, J. R. Kitchin, E. Leonhard Kolsbjerg, J. Kubal, K. Kaasbjerg, S. Lysgaard, J. Bergmann Maronsson, T. Maxson, T. Olsen, L. Pastewka, A. Peterson, C. Rostgaard, J. Schiøtz, O. Schütt, M. Strange, K. S. Thygesen, T. Vegge, L. Vilhelmsen, M. Walter, Z. Zeng, K. W. Jacobsen, The atomic simulation environment—A Python library for working with atoms. *J. Phys. Condens. Matter* **29**, 273002 (2017).

58. J. Behler, M. Parrinello, Generalized neural-network representation of high-dimensional potential-energy surfaces. *Phys. Rev. Lett.* **98**, 146401 (2007).

59. A. Reinhardt, M. Bethkenhagen, F. Coppari, M. Millot, S. Hamel, B. Cheng, Thermodynamics of high-pressure ice phases explored with atomistic simulations. *Nat. Commun.* **13**, 4707 (2022).

60. A. P. Thompson, H. M. Aktulga, R. Berger, D. S. Bolintineanu, W. M. Brown, P. S. Crozier, P. J. in ‘t Veld, A. Kohlmeyer, S. G. Moore, T. D. Nguyen, R. Shan, M. J. Stevens, J. Tranchida, C. Trott, S. J. Plimpton, LAMMPS—A flexible simulation tool for particle-based materials modeling at the atomic, meso, and continuum scales. *Comput. Phys. Commun.* **271**, 108171 (2022).

61. G. Kresse, J. Hafner, Ab initiomolecular-dynamics simulation of the liquid-metal-amorphous-semiconductor transition in germanium. *Phys. Rev. B* **49**, 14251–14269 (1994).

62. G. Kresse, J. Furthmüller, Efficient iterative schemes forab initiototal-energy calculations using a plane-wave basis set. *Phys. Rev. B* **54**, 11169–11186 (1996).

63. G. Kresse, J. Furthmüller, Efficiency of ab-initio total energy calculations for metals and semiconductors using a plane-wave basis set. *Comput. Mater. Sci.* **6**, 15–50 (1996).

64. J. P. Perdew, K. Burke, M. Ernzerhof, Generalized gradient approximation made simple. *Phys. Rev. Lett.* **77**, 3865–3868 (1996).

65. R. Nelson, C. Ertural, J. George, V. L. Deringer, G. Hautier, R. Dronskowski, LOBSTER: Local orbital projections, atomic charges, and chemical-bonding analysis from projector-augmented-wave-based density-functional theory. *J. Comput. Chem.* **41**, 1931–1940 (2020).

66. S. P. Ong, W. D. Richards, A. Jain, G. Hautier, M. Kocher, S. Cholia, D. Gunter, V. L. Chevrier, K. A. Persson, G. Ceder, Python Materials Genomics (pymatgen): A robust, open-source python library for materials analysis. *Comput. Mater. Sci.* **68**, 314–319 (2013).

67. B. Morgan, vasppy (version 0.7.1.0) software. python package (2021); https://pypi.org/project/vasppy/0.7.1.0/.

68. A. R. McCluskey, S. W. Coles, B. J. Morgan, Accurate estimation of diffusion coefficients and their uncertainties from computer simulation. *J. Chem. Theory Comput.* **21**, 79–87 (2025).

69. A. R. McCluskey, A. G. Squires, J. Dunn, S. W. Coles, B. J. Morgan, kinisi: Bayesian analysis of mass transport from molecular dynamics simulations. *J. Open Source Softw.* **9**, 5984 (2024).

70. C. R. Harris, K. J. Millman, S. J. van der Walt, R. Gommers, P. Virtanen, D. Cournapeau, E. Wieser, J. Taylor, S. Berg, N. J. Smith, R. Kern, M. Picus, S. Hoyer, M. H. van Kerkwijk, M. Brett, A. Haldane, J. F. del Río, M. Wiebe, P. Peterson, P. Gérard-Marchant, K. Sheppard, T. Reddy, W. Weckesser, H. Abbasi, C. Gohlke, T. E. Oliphant, Array programming with NumPy. *Nature* **585**, 357–362 (2020).

71. K. D. Fong, B. Sumić, N. O'Neill, C. Schran, C. P. Grey, A. Michaelides, The interplay of solvation and polarization effects on ion pairing in nanoconfined electrolytes. *Nano Lett.* **24**, 5024–5030 (2024).

72. C. Donati, J. F. Douglas, W. Kob, S. J. Plimpton, P. H. Poole, S. C. Glotzer, Stringlike cooperative motion in a supercooled liquid. *Phys. Rev. Lett.* **80**, 2338–2341 (1998).

73. B. Morgan, bjmorgan/dataargyroditedisorder: Manuscript Resubmission Release, version 1.0, Zenodo (2020); https://zenodo.org/record/4338578.

74. S. W. Coles, A. Hajibabaei, V. Kapil, X. R. Advincula, C. Schran, S. J. Cox, A. Michaelides, Research data supporting: "Nanoconfined superionic water is a molecular superionic" (2026); https://doi.org/10.17863/CAM.127350.

75. I. Batatia, D. P. Kovacs, G. N. C. Simm, C. Ortner, G. Csanyi, "MACE: Higher order equivariant message passing neural networks for fast and accurate force fields," in *Advances in Neural Information Processing Systems*, A. H. Oh, A. Agarwal, D. Belgrave, K. Cho, Eds. (2022); https://openreview.net/forum?id=YPpSngE-ZU.

76. A. Hajibabaei, W. J. Baldwin, G. Csányi, S. J. Cox, Symmetry breaking in the superionic phase of silver iodide. *Phys. Rev. Lett.* **134**, 026306 (2025).

77. S. Di Pino, Y. A. Perez Sirkin, U. N. Morzan, V. M. Sánchez, A. Hassanali, D. A. Scherlis, Water self-dissociation is insensitive to nanoscale environments. *Angew. Chem. Int. Ed. Engl.* **62**, e202306526 (2023).

78. D. Muñoz-Santiburcio, D. Marx, Confinement-controlled aqueous chemistry within nanometric slit pores: Focus review. *Chem. Rev.* **121**, 6293–6320 (2021).

79. M. French, T. R. Mattsson, R. Redmer, Diffusion and electrical conductivity in water at ultrahigh pressures. *Phys. Rev. B* **82**, 174108 (2010).
